# Pediatric healthcare service utilization after the end of COVID-19 state of emergency in Northern Italy: a 6-year quasi-experimental study using interrupted time-series analysis

**DOI:** 10.3389/fpubh.2025.1575047

**Published:** 2025-08-21

**Authors:** Matteo Puntoni, Caterina Caminiti, Giuseppe Maglietta, Marcello Lanari, Giacomo Biasucci, Agnese Suppiej, Federico Marchetti, Alessandro De Fanti, Fabio Caramelli, Lorenzo Iughetti, Chiara Ghizzi, Enrico Valletta, Gianluca Vergine, Marcello Stella, Beatrice Campana, Valentina Fainardi, Michela Deolmi, Susanna Esposito, Emilia-Romagna Paediatric COVID-19 Network

**Affiliations:** ^1^Clinical and Epidemiological Research Unit, University-Hospital of Parma, Parma, Italy; ^2^Pediatric Emergency Unit, IRCCS Azienda Ospedaliera Universitaria di Bologna, Bologna, Italy; ^3^Pediatrics and Neonatology Unit, Guglielmo da Saliceto Hospital, Piacenza, Italy; ^4^Department of Medicine and Surgery, University of Parma, Parma, Italy; ^5^Pediatric Clinic, University of Ferrara, Ferrara, Italy; ^6^Pediatrics and Neonatology Unit, Ravenna Hospital, AUSL Romagna, Ravenna, Italy; ^7^Paediatrics Unit, Santa Maria Nuova Hospital, AUSL-IRCCS of Reggio Emilia, Reggio Emilia, Italy; ^8^Pediatric Intensive Care Unit, IRCCS Azienda Ospedaliera Universitaria di Bologna, Bologna, Italy; ^9^Pediatrics Unit, Department of Medical and Surgical Sciences of Mothers, Children and Adults, University of Modena and Reggio Emilia, Modena, Italy; ^10^Paediatrics Unit, Maggiore Hospital, Bologna, Italy; ^11^Pediatric Unit, G.B. Morgagni—L. Pierantoni Hospital, AUSL Romagna, Forlì, Italy; ^12^Pediatric Clinic, Rimini Hospital, AUSL Romagna, Rimini, Italy; ^13^Pediatric Unit, AUSL Romagna, Cesena, Italy; ^14^Pediatric Clinic, University-Hospital of Parma, Parma, Italy

**Keywords:** healthcare service utilization, post-emergency, post-pandemic effects, pediatric hospitalizations, pediatric emergency department, mental disorders, quasi-experimental design, interrupted time series regression analysis

## Abstract

**Background:**

Evidence exists on the major disruptions in pediatric healthcare services occurred during the COVID-19 pandemic, but what happened when all restrictions were lifted is unclear. This study examined trends in pediatric hospital admission and Emergency Department visit rates during the first 12 months following the end of the state of emergency in Italy, compared to pre-pandemic levels.

**Methods:**

We conducted a multicenter, retrospective, quasi-experimental before after study including 11 North Italian hospitals. Using electronic health records from March 2017 to March 2023, we compared standardized rates recorded during 1 year following the end of the emergency with the situation before the pandemic, using interrupted time series. We examined trends overall and for individual diagnostic categories that had exhibited the largest impact in our previous studies concerning data up to March 2022.

**Results:**

A total of 104,083 hospitalizations and 858,762 Pediatric Emergency Department visits were analyzed. Compared to the 3 years before the outbreak, post-emergency hospitalization rates increased by 23% (Standardized Hospitalization Rate Ratio 1.23, 95% CI 1.20–1.25), whereas Pediatric Emergency Department visits after a sharp decrease returned to pre-pandemic rates (Standardized Incidence Rate Ratio 0.98, 95% CI 0.96–1.00). Mental health-related hospitalizations exhibited the largest increase, peaking in the first months of the post-pandemic year (level change, Hospitalization Rate Ratio (HRR)2.57, 95%CI 1.61–4.12), then decreasing slightly in the last months but still maintaining much higher than pre-pandemic values. Notably, hospitalization rates in adolescent girls (12–17 years) increased almost 4-fold (level change, HRR 3.72, 95%CI 2.02–6.85, *p* < 0.001), whereas the increase was not significant for boys in the same age group (level change, HRR 1.42, 95%CI 0.65–3.11, *p* = 0.378). Respiratory diseases, after drastically declining during the two pandemic years, experienced steadily increasing monthly trends, finally stabilizing in the post-pandemic year at pre-pandemic levels.

**Conclusion:**

The COVID-19 pandemic has had long-term consequences on pediatric healthcare utilization. Data from this and future studies can guide the development of proactive policies aiming to mitigate healthcare disruptions and ensure access to essential pediatric services in the event of future health crises, with special consideration to vulnerable populations. The persistent rise in hospital admissions for mental health disorders, particularly teenage girls, make this field a challenging, absolute priority for public health.

## 1 Introduction

Children and adolescents were relatively spared by the Severe Acute Respiratory Syndrome Coronavirus-2 (SARS-CoV-2); they rarely contracted the virus, and when infected, they often experienced only mild or no symptoms at all (1). As a result, healthcare systems redirected their focus to managing the overwhelming surge of adult COVID-19 patients (2), rapidly reshaping hospital infrastructures, postponing or canceling non-urgent visits, closing non-COVID-19 wards (3, 4), and even repurposing children’s hospitals to accommodate adult COVID-19 treatment units (5–7). Furthermore, the restrictive measures enforced globally to contain viral spread, such as lockdowns and shutdown of activities, often referred to as Non-Pharmaceutical interventions (NPIs), also had important, unintended, indirect consequences on pediatric healthcare provision, as well as on parents’ medical seeking behavior (8, 9). A systematic review (10), which included data from 69 studies and approximately 14 million individual visits, reported a significant reduction in paediatric emergency department (PED) visits of 63.86% (95% CI 60.40–67.31%), with the largest decrease in low-acute patients. Teasdale et al. (11), in a cross-sectional online survey of 2074 US parents of children ≤12 years, reported that in 2021, 41.3% (95%CI 38.3–43.8) of children missed a routine health visit, and a third of parents (33.1%; 95%CI 30.7–35.5) reported their child had missed a vaccination. Importantly, NPIs, especially school closure and social distancing, also directly affected young people, substantially modifying their lifestyle, causing isolation and disturbances to their routine, and producing psychosocial consequences, all of which may have influenced their healthcare needs (12, 13).

Despite the relevance of these phenomena, the available evidence mostly concerns specific medical conditions or care settings, and/or with limited follow-up after NPI lifting (8, 14, 14–). Thus, there is a lack of comprehensive information on what happened to the use of paediatric health services after the end of the state of emergency and the removal of restrictions. It is essential to know whether the pre-pandemic situation was fully restored, whether a recovery period occurred, or whether the pandemic led to lasting changes, e.g., a reduction in the use of unnecessary hospital services. The present study attempted to fill this gap, building on our previous work in this area. In two recently published studies, we have quantified the impact of NPIs on trends in paediatric hospital admissions (15) and paediatric emergency department (PED) attendance (16), respectively. Both studies were conducted in 12 centres in Northern Italy and used a rigorous Interrupted Time Series (ITS) regression analysis to compare a 3-year pre-pandemic versus a 2-year pandemic emergency period. The results showed that, compared to the pre-pandemic years, statistically significant decreases of hospitalization and PED attendance rates occurred during school closure (35 and 58%, respectively) and in the subsequent 18 months in which mitigation measures were applied (19 and 39%, respectively). In both settings, the greatest reduction was observed in “Respiratory Diseases” for nearly the entire pandemic period. In contrast, the category “Mental Disorders” experienced a sharp decline only during the first two months of lockdown, followed by steadily increasing rates throughout the emergency period, ultimately reaching levels well above pre-pandemic values. In our previous work on PED care, we examined the trend in rates for two additional diagnostic categories, “Symptoms, Signs, and Ill-defined Conditions” and “Injuries and Poisonings,” as they were highly prevalent and accounted for the majority of the decline observed during the pandemic. In both of our previous studies, we concluded by emphasizing the need to further investigate the effects on health services utilization following the pandemic emergency. This would provide additional evidence to support policy decisions and offer guidance to citizens in the event of future pandemic crises.

To this end, we report herein the analysis of trends in pediatric hospitalizations and PED visits—both overall and for the most impacted diagnostic categories—during the year following the state of emergency in Italy. To our knowledge, this is the first study that examines the impact of the pandemic on both care settings over an extended follow-up period, applying ITS analysis.

## 2 Materials and methods

### 2.1 Study design and setting

This multicenter, retrospective, quasi-experimental controlled before-after study aimed to estimate changes in pediatric healthcare utilization (hospital admissions and PED attendance) following the end of the COVID-19 state of emergency in Northern Italy, compared to the period of NPI implementation and to the situation before the pandemic. The setting and methodology of this study have been described previously (15, 16).

Along with the overall analysis of rate trends, we also report on four diagnostic categories that, based on our previous studies, appeared to have been most impacted by pandemic restrictions, showing considerable changes in healthcare utilization.

The present study spanned from March 2017 to March 2023, defining the implementation of NPIs as an intervention event.

### 2.2 Intervention

As in our previous two studies, we used the beginning of NPI implementation in Italy (national lockdown declared in March 2020) as delimitation. The preceding 3 years constituted the pre-COVID-19 phase (PC), followed by a School Closure phase (SC, from March to September 2020), and by a mitigation measures phase (MM, October 2020 to March 2022), when schools were reopened but milder restrictions remained. This work adds and focuses on the Post Emergency phase (PE), spanning from the end of the state of emergency (declared in Italy on March 31^,^ 2022) to March 2023. In PE, all NPIs were lifted.

### 2.3 Participants

This analysis comprised data from 11 of the 15 (73%) hospitals in the Emilia-Romagna Region (one of the 12 participating centers in the first two studies did not provide updates). Emilia-Romagna was one of the first and most severely affected regions by the COVID-19 pandemic, facing high mortality, healthcare strain, and strict social restrictions. This context makes the analyzed sample particularly suitable for assessing the pandemic’s impact on healthcare utilization for non-COVID conditions in children.

### 2.4 Data sources

Study data were anonymously gathered from routine electronic clinical records contained in the databases of the Emilia-Romagna Regional Health Trust (hospital discharge forms and emergency care visit registry). Collected data included: age, sex, admission and discharge dates, and diagnoses coded using the International Classification of Diseases, Ninth Revision, Clinical Modification (ICD-9-CM).

### 2.5 Statistical analysis

The statistical methods have been detailed in our previous papers (15, 16). Briefly, we considered the monthly frequency of hospitalizations and of PED attendance, total and for ICD9-CM categories (the first three characters), during the study period. We calculated standardized hospitalization rates (SHR) and, for PED attendance, standardized incidence rates (SIR) per 100,000 person-month considering as standard the resident population in Europe in 2020, adjusting for age and sex. For each considered diagnostic category, we measured how any of the times changed with respect to the pre-COVID-19 (PC) phase. We estimated the Standardized Hospitalization Rate Ratios (SHRR) and, for PED attendance, the Standardized Incidence Rate Ratios (SIRR), and their 95% Confidence Intervals (95% CI). To investigate changes in the different periods, interrupted time series (ITS) regression analysis was used. This segmented approach allows to estimate changes attributable to an intervention, in terms of overall (as time trend), immediate (as changes in level) and sustained (increase or decrease in the slope) effects, while accounting for pre-intervention secular trends. Additionally, in order to determine the rate variation in each individual phase independently from the other phases, we estimated the time coefficients using deseasonalized ITS regression models. All statistical analyses were centralized and performed with STATA (StataCorp. 2023. Stata Statistical Software: Release 18. College Station, TX: StataCorp LLC.).

## 3 Results

The analysis concerned 104,083 hospitalizations and 858,762 PED visits, recorded in the 11 participating centers from March 1^st^, 2017, to March 31^st^, 2023. Overall, in the 3 post-pandemic years compared to the 3 pre-pandemic years, there was a decrease of 4,323 (−8%) hospitalizations and 153,980 (−30%) PED visits. The demographic characteristics of the analyzed sample in the four phases are reported in Table 1. Overall, patients were predominantly male, in both the pediatric hospitalization and emergency department settings (57.2 and 56.3%, respectively). Hospital admissions mainly concerned infants 0 to 1 year old (36.6%), while visits to the PED were equally distributed among the different age groups. The standardized annual utilization rates, also shown in Table 1, pointed to a different behavior in the year following the state of emergency (PE) in the two settings. Specifically, compared to pre-pandemic levels, hospital admissions exhibited an important increase (SHR 4.606 vs. 3.759×100,000 person-year), whereas the PED attendance rate was very similar (SIR 2,836 vs. 2.897 × 100,000 person-month). Supplementary Tables S1 and S2 show the distribution of major disease categories among all pediatric hospitalizations and PED attendance rates, with respiratory diseases accounting for 17.2 and 14.5% of admissions, respectively.

**Table 1:** Characteristics of the analyzed sample, presented separately for hospitalizations and PED attendance, across the four phases.

	PC (Mar 1, 2017–Feb 28, 2020)		SC (Mar 1, 2020–Sep 30, 2020)		MM (Oct 1, 2020–Mar 31, 2022)		PE (Apr 1, 2022–Mar 31, 2023)		Whole period (Mar 1, 2017–Mar 31, 2023)	
Hospitalizations	*n* = 54,218		*n* = 6,737		*n* = 21,973		*n* = 21,155		*N* = 104,083	
Sex, *n* (%) males	31,019	(57.2)	3,786	(56.2)	12,487	(56.8)	12,280	(58.1)	59,572	(57.2)
Age class, y *n* (%)										
0–1	20,276	(37.4)	2,694	(40.0)	8,147	(37.1)	7,010	(33.1)	38,127	(36.6)
2–5	13,527	(25.0)	1,325	(19.7)	4,666	(21.2)	5,395	(25.5)	24,913	(23.9)
6–11	9,173	(16.9)	1,180	(17.5)	3,625	(16.5)	3,582	(16.9)	17,560	(16.9)
12–17	11,242	(20.7)	1,538	(22.8)	5,535	(25.2)	5,168	(24.4)	23,483	(22.6)
Standardized Hospitalization Rates, SHR (95%CI)*	3,759 (3,707-3,811)		2,451 (2,408-2,494)		3,162 (3,113-3,211)		4,606 (4,548-4,665)		3,653 (3,601-3,705)	
PED attendance	*n* = 506,246		*n* = 41,714		*N* = 153,425		*N* = 157,127		*N* = 858,762	
Sex, *n* (%) males	283,806	(56.0)	23,896	(57.3)	86,793	(56.6)	88,894	(56.5)	483,389	(56.3)
Age class, y *n* (%)										
0–1	117,784	(23.3)	8,962	(21.5)	35,063	(22.8)	34,377	(21.9)	196,186	(22.9)
2–5	149,871	(29.6)	10,319	(24.7)	42,045	(27.4)	47,379	(30.2)	249,614	(29.1)
6–11	136,268	(26.9)	11,975	(28.7)	38,887	(25.4)	42,506	(27.1)	229,636	(26.7)
12–17	102,573	(20.2)	10,458	(25.1)	37,430	(24.6)	32,865	(20.9)	183,326	(21.4)
Standardized Incidence Rates, SIR (95%CI)^*^	2,897 (2,850-2,944)		1,238 (1,207-1,269)		1,811 (1,773-1,849)		2,836 (2,788-2,883)		2,481 (2,437-2,525)	

PED, Paediatric Emergency Department; PC, pre-COVID19 phase; SC, School closure phase; MM, Mitigation measures phase; PE, Post-emergency phase; SHR, Standardized Hospitalization Rate; SIR, Standardized Incidence Rate.

* Rates are standardized × 100,000 (pop EU 2020) and adjusted for age and sex.

**Figure 1:**
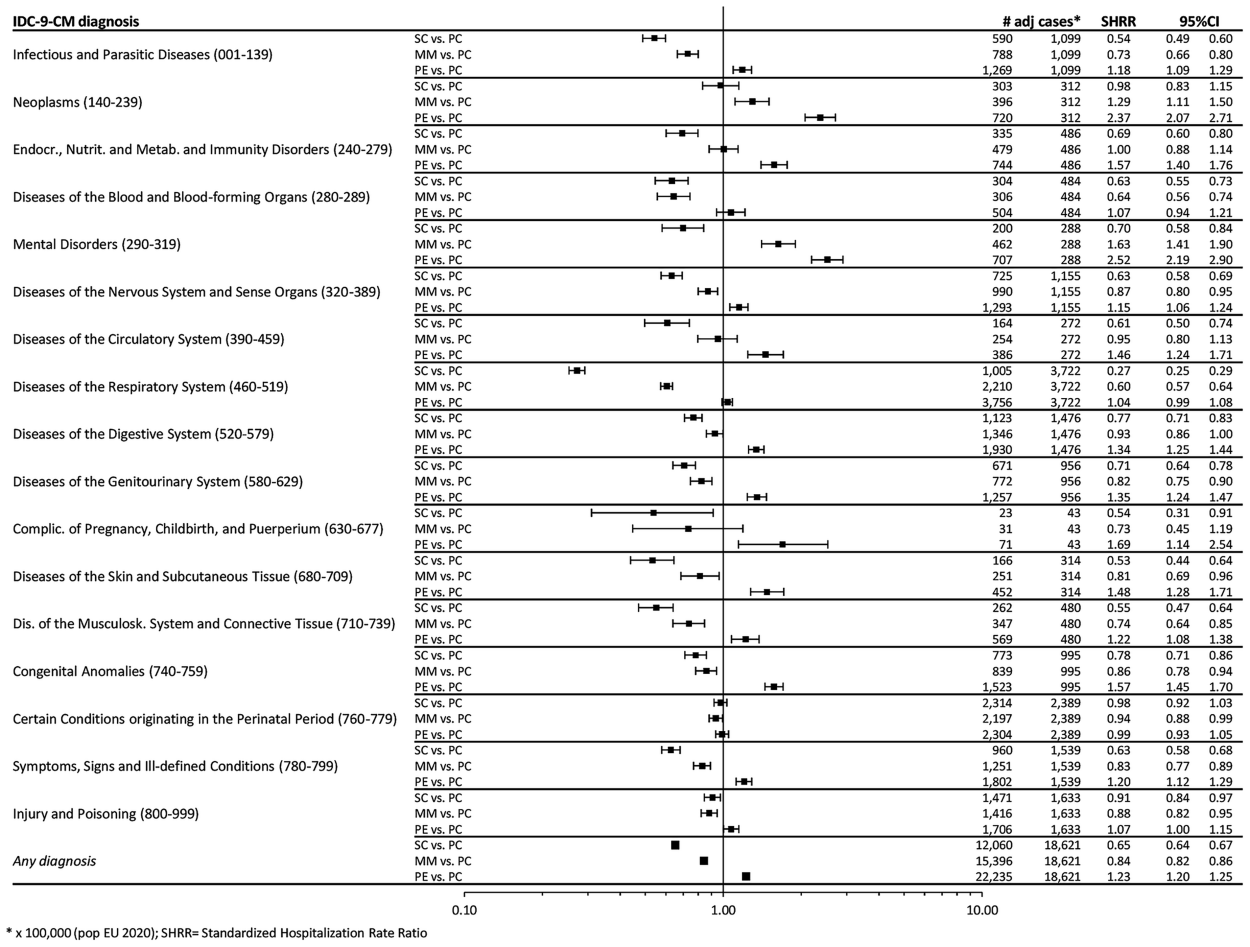
Forest plot of Standardized Hospitalization Rate Ratios (SHRR). Note: Estimates are reported as × 100,000 person-year and are age & sex standardized using as Standard the European resident population in 2020. SHR, Standardized Hospitalization Rate; PC, Pre-COVID19 phase; SC, School Closure phase; PE, Post-Emergency phase.

**Figure 2:**
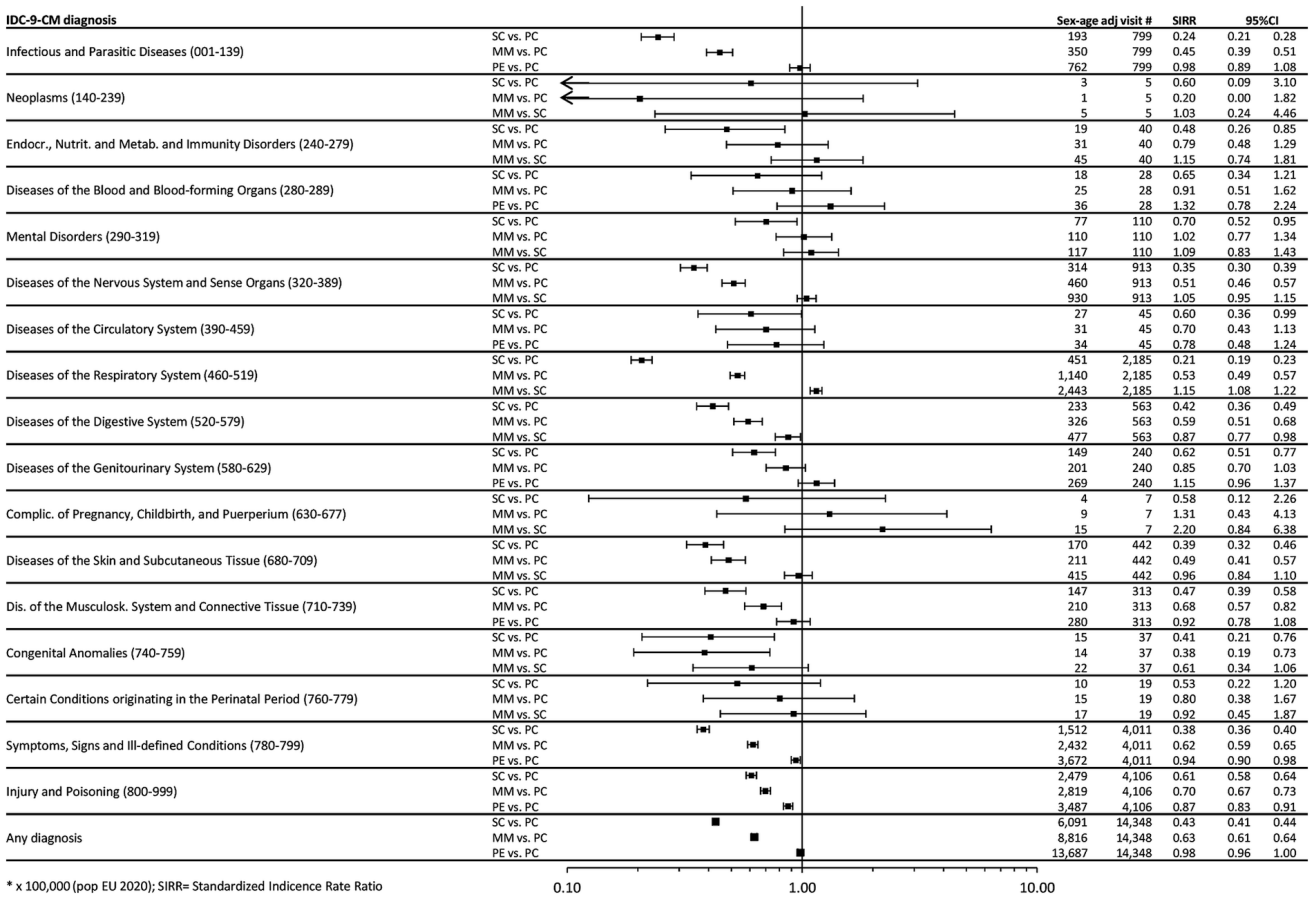
Forest plot of Standardized Incidence Rate Ratios (SIRR). Note: Estimates are reported as × 100,000 person-month and are age & sex standardized using as Standard the European resident population in 2020. SIRR, Standardized Incidence Rate Ratio; PC, Pre-COVID19 phase; SC, School Closure phase; PE, Post-Emergency phase.

To investigate changes over time, we compared rates documented after the beginning of the pandemic for each of the three considered phases (SC, MM and PE) with those from the pre-pandemic phase (PC). Concerning hospitalizations, Figure 1 shows comparisons in terms of SHRR, collectively and for individual ICD9-CM categories. Overall, following the relevant reductions reported in SC (−35%, SHRR 0.65, 95%CI 0.64–0.67) and MM (−16%, SHRR 0.84, 95%CI 0.82–0.86), in PE a statistically significant 23% increase was observed (SHRR 1.23, 95%CI: 1.20–1.25), distributed to varying degrees across all diagnostic categories. In particular, “Mental Disorders” exhibited the most considerable increase, with a rate in PE being two and a half times higher than in PC (SHRR 2.52, 95%CI 2.19–2.90). Concerning PED attendance, we conducted the same comparisons (each of three pandemic phases vs. pre-pandemic phase) in terms of SIRR (Figure 2). In this setting, after a drastic reduction seen in SC (−57%, SIRR 0.43, 95%CI 0.41–0.44) and MM (−37%, SIRR 0.63, 95%CI 0.61–0.64), the rate returned to PC levels, overall (SIRR 0.98, 95%CI 0.96–1.00) and generally for all diagnostic categories. Only “Respiratory Diseases” experienced a significant 15% increase (SIRR 1.15, 95%CI 1.08–1.22) in the year following the pandemic emergency.

To further investigate the impact of the pandemic on post-emergency healthcare utilization, we present below the results of the ITS regression analysis, conducted for any diagnosis and for the four diagnostic categories that in our previous studies have been shown to be most impacted during the emergency.

### 3.1 Hospitalization and PED attendance for all diagnostic categories

**Figure 3:**
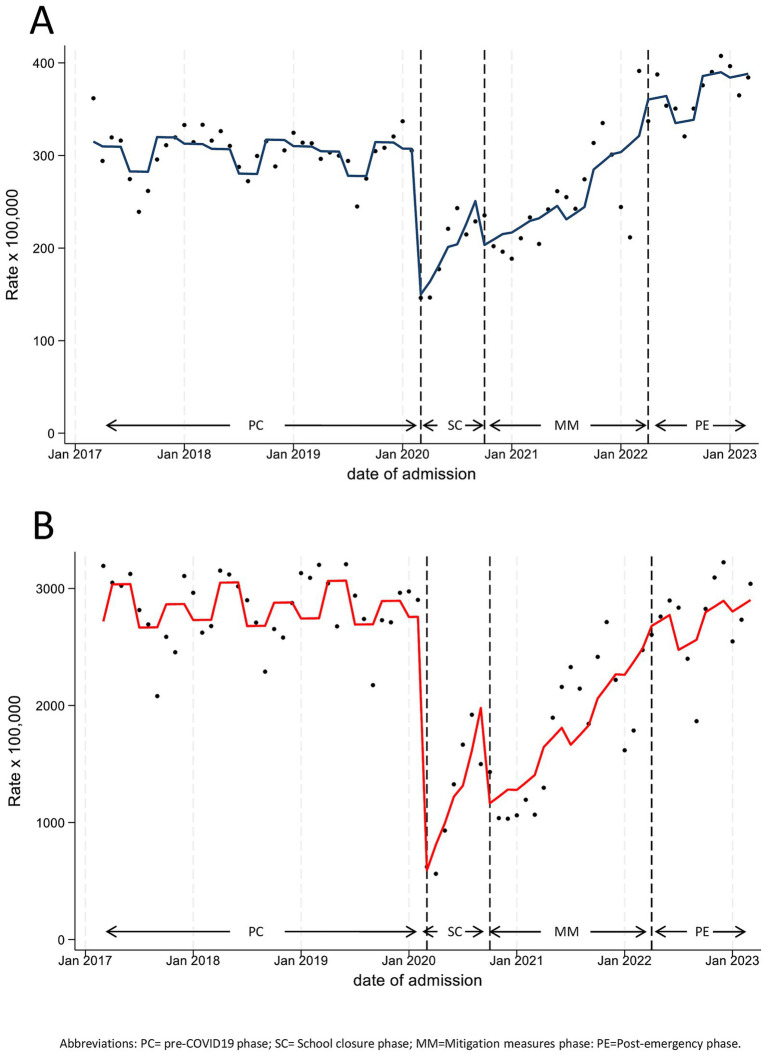
Monthly hospitalization **(A)** and Pediatric Emergency Department Incidence **(B)** Rate for any disease, with line trend from ITS regression analysis. PC, Pre-COVID19 phase; SC=School Closure phase; MM, Mitigation Measures phase; PE, Post-Emergency phase.

Model estimates from ITS are shown in Figure 3 and in Table 2. As reported in our previous papers, the two emergency phases (SC and MM) both commenced with significant decreases, followed by increasing monthly trends, both for hospital admissions and PED visits. In both settings, rates returned to pre-pandemic levels only in autumn 2021, 18 months after the beginning of the pandemic. In the post-emergency period, trends increased slightly, although not significantly, in both settings compared to PC (slope change: for hospitalizations, 1% per month, Hospitalization Rate Ratio (HRR) 1.01, 95%CI 0.99–1.02; for PED attendance, 2% per month, Incidence Rate Ratio (IRR) 1.02, 95%CI 0.99–1.04). However, while hospital admission rates significantly exceeded pre-pandemic levels (level change, +21%, HRR 1.21, 95%CI 1.02–1.43, p 0.030), the PED attendance seems to stabilize at PC values (level change, −15%, IRR 0.85, 95%CI 0.66–1.10). Noteworthy, within PE we observed a significant 1% monthly variation of the hospitalization rate (HRR, 1.01, 95%CI 1.00–1.02), which did not occur in the pre-pandemic period (HRR, 1.00, 95%CI 0.99–1.01; Supplementary Table S3).

**Table 2:** Interrupted time-series analysis results on hospitalizations and PED attendance rates.

Variable			
Hospitalizations	HRR	95%CI	*p*-Value
Level change^a^			
SC vs. PC	0.44	0.36–0.54	<0.001
MM vs. PC	0.63	0.56–0.72	<0.001
PE vs. PC	1.21	1.02–1.43	0.030
Slope change^b^			
SC vs. PC	1.11	1.06–1.16	<0.001
MM vs. PC	1.03	1.02–1.04	<0.001
PE vs. PC	1.01	0.99–1.02	0.429
Time trend^c^	1.00	0.99–1.01	0.623
Season			
Summer	1.00		
Winter	1.12	1.04–1.19	0.002
Spring	1.09	1.03–1.17	0.006
Autumn	1.13	1.06–1.21	<0.001

PED attendance	IRR	95%CI	*p*-value
Level change^a^			
SC vs. PC	0.18	0.12–0.26	<0.001
MM vs. PC	0.38	0.31–0.47	<0.001
PE vs. PC	0.85	0.66–1.10	0.208
Slope change^b^			
SC vs. PC	1.23	1.13–1.33	<0.001
MM vs. PC	1.05	1.03–1.06	<0.001
PE vs. PC	1.02	0.99–1.04	0.170
Time trend^c^	1.00	0.99–1.01	0.848
Season			
Summer	1.00		
Winter	1.02	0.93–1.13	0.672
Spring	1.14	1.04–1.25	0.007
Autumn	1.07	0.98–1.18	0.147

PED, Pediatric Emergency Department; HRR, Hospitalization Rate Ratio; IRR, incidence rate ratio; PC, pre-COVID19 phase; SC, School closure phase; MM, Mitigation measures phase; PE, Post-emergency phase; 95%CI: 95% confidence interval. Pseudo R2 for hospitalization rate model = 0.72, for PED attendance rate model = 0.83.

a Level change refers to an abrupt level change of the Incidence rate between the phases.

b Slope change refers to slope change of the incidence rate over time between the phases.

c Time trend refers to the change of Incidence rate associated with a time unit increase.

### 3.2 Focus on diagnostic categories highlighted in our previous studies

#### 3.2.1 Mental disorders

**Figure 4:**
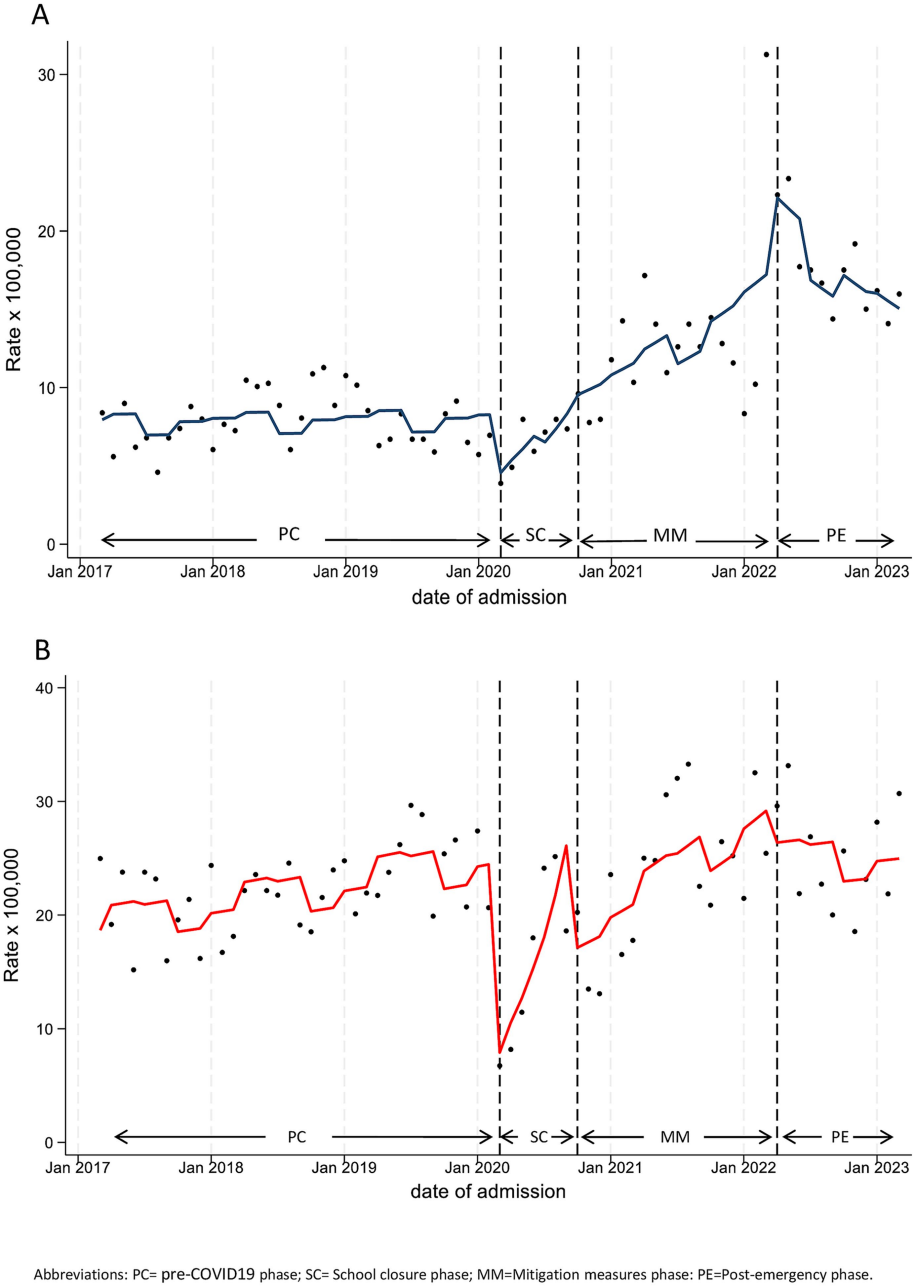
Monthly hospitalization **(A)** and Pediatric Emergency Department Incidence **(B)** Rate for Mental Disease, with line trend from ITS regression analysis. PC, pre-COVID19 phase; SC=School closure phase; MM, Mitigation measures phase; PE, post-emergency phase.

From the analysis of our previous two studies, it was evident that, for this diagnostic category, the behavior of rates during the pandemic exhibited substantially different trends compared to the overall trend and to that of other categories. In particular, although a significant reduction in rates was observed in both settings at the beginning of lockdown (level change: for hospitalizations HRR 0.49, 95%CI 0.27–0.89; for PED attendance IRR 0.27, 95%CI 0.17–0.42), pre-COVID19 levels were restored starting from the last months of SC and continued to rise during MM (slope change: for hospitalizations, 3% per month, HRR 1.03, 95%CI 1.01–1.06; for PED attendance, 2% per month, IRR 1.02, 95%CI 1.00–1.04). This constant increase led to monthly rates with values higher than pre-pandemic ones already during MM, in both settings (Figure 4; Table 3).

**Table 3:** Interrupted time-series analysis results on hospitalizations and PED attendances rates for mental disorders.

Variable			
Hospitalizations	HRR	95%CI	*p*-value
Level change^a^			
SC vs. PC	0.49	0.27–0.89	0.018
MM vs. PC	1.14	0.82–1.57	0.441
PE vs. PC	2.57	1.61–4.12	<0.001
Slope change^b^			
SC vs. PC	1.13	1.00–1.27	0.047
MM vs. PC	1.03	1.01–1.06	0.003
PE vs. PC	0.97	0.94–1.00	0.081
Time trend^c^	1.00	0.99–1.01	0.803
Season			
Summer	1.00		
Winter	1.15	0.96–1.37	0.130
Spring	1.20	1.01–1.42	0.040
Autumn	1.12	0.94–1.33	0.198

PED attendances	IRR	95%CI	*p*-value
Level change^a^			
SC vs. PC	0.27	0.17–0.42	<0.001
MM vs. PC	0.69	0.54–0.87	0.002
PE vs. PC	0.80	0.57–1.12	0.191
Slope change^b^			
SC vs. PC	1.19	1.10–1.30	<0.001
MM vs. PC	1.02	1.00–1.04	0.022
PE vs. PC	1.00	0.97–1.03	0.838
Time trend^c^	1.01	1.00–1.01	0.008
Season			
Summer	1.00		
Winter	0.92	0.82–1.04	0.185
Spring	1.02	0.90–1.15	0.745
Autumn	0.87	0.77–0.98	0.021

PED, Pediatric Emergency Department; HRR, Hospitalization Rate Ratio; IRR, incidence rate ratio; PC, pre-COVID19 phase; PC, pre-COVID19 phase; SC, School closure phase; MM, Mitigation measures phase; PE, Post-emergency phase; 95%CI: 95% confidence interval. Pseudo R2 for hospitalization rate model = 0.45, for PED attendance rate model = 0.26.

a Level change refers to an abrupt level change of the Incidence rate between the phases.

b Slope change refers to slope change of the incidence rate over time between the phases.

c Time trend refers to the change of Incidence rate associated with a time unit increase.

Trend analysis of PED attendance rates after the pandemic emergency (PE vs. PC) did not highlight any significant changes (slope change, IRR 1.00, 95%CI 0.97–1.03) and suggests stabilization at pre-pandemic values. Conversely, hospital admission rates reached their peak in the initial few months of PE (level change, HRR 2.57, 95%CI 1.61–4.12), followed by a monthly decrease (slope change, −3% per month, HRR 0.97, 95%CI 0.94–1.00), which is close to statistical significance (*p* = 0.081). Yet, although hospitalization rates in the last months of PE were much lower than in the first months, the recorded values at the end of PE remained much higher than pre-pandemic ones. In fact, as reported in Supplementary Table S3, the hospitalization rate in the year following the end of the emergency significantly dropped by 3% monthly (HRR, 0.97, 95%CI 0.95–0.99), which did not occur in the pre-pandemic period (HRR, 1.00, 95%CI 0.99–1.01).

**Figure 5:**
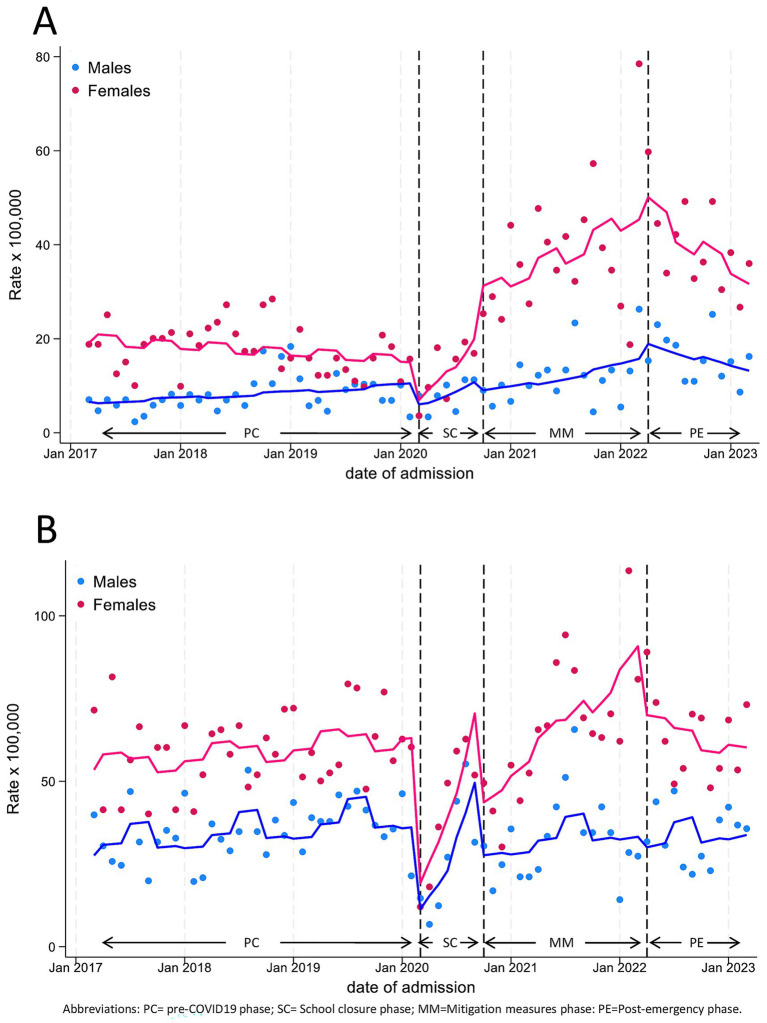
Monthly hospitalization **(A)** and Pediatric Emergency Department Incidence **(B)** Rate for Mental Disease, by sex, in adolescents (age 12–17 years), with line trend from ITS regression analysis. PC, pre-COVID19 phase; SC, School closure phase; MM, Mitigation measures phase; PE, post-emergency phase.

Subgroup analyses in our previous work highlighted sex and age differences in both hospitalization and PED attendance rates, which we confirmed for the post-emergency year (Supplementary Tables S4–S8, Supplementary Figures S1, S2). In particular, the hospitalization rates in the adolescent subgroup (12–17 years), in PE vs. PC increased by nearly 4 times for females (level change, HRR 3.72, 95%CI 2.02–6.85, *p* < 0.001), while the increase was not significant for males (level change, HRR 1.42, 95%CI 0.65–3.11, *p* = 0.378; Table 4; Figure 5).

**Table 4:** Interrupted time-series analysis results on hospitalization rates for Mental disorders, in adolescents (12-17y), by sex.

	Males			Females		
	HRR	95%CI	*p*-value	HRR	95%CI	*p*-value
Level change^a^						
SC vs. PC	0.52	0.21–1.27	0.151	0.39	0.17–0.91	0.030
MM vs. PC	0.75	0.44–1.29	0.297	1.96	1.31–2.91	0.001
PE vs. PC	1.42	0.65–3.11	0.378	3.72	2.02–6.85	<0.001
Slope change^b^						
SC vs. PC	1.10	0.92–1.31	0.310	1.20	1.02–1.42	0.029
MM vs. PC	1.02	0.98–1.06	0.290	1.03	1.01–1.06	0.007
PE vs. PC	0.95	0.89–1.01	0.107	0.97	0.93–1.02	0.262
Time trend^c^	1.01	1.00–1.03	0.077	0.99	0.98–1.01	0.237
Season						
Summer	1.00			1.00		
Winter	1.06	0.79–1.42	0.685	1.02	0.82–1.26	0.882
Spring	1.00	0.75–1.34	0.997	1.12	0.91–1.38	0.286
Autumn	1.07	0.81–1.42	0.627	1.11	0.90–1.36	0.331

HRR, Hospitalization Rate Ratio; PC, Pre-COVID19 phase; SC, School closure phase; MM, Mitigation Measures phase; PE, Post Emergency phase; 95%CI: 95% confidence interval. Pseudo R2 = 0.16 for Males model, 0.41 for Females model.

a Level change refers to an abrupt level change of the Incidence rate between the periods.

b Slope change refers to slope change of the incidence rate over time between the periods.

c Time trend refers to the change of Incidence rate associated with a time unit increase.

#### 3.2.2 Disease of the respiratory system

In both settings, rates for this diagnostic category experienced the sharpest decline among all categories during SC, followed by increasing monthly trends until the end of MM, ultimately stabilizing in PE at rates comparable to the pre-pandemic period (Supplementary Figure S3; Supplementary Table S9). In fact, both slope changes were positive but not statistically significant (slope change: for hospitalizations, 5% per month, HRR 1.05, 95%CI 1.00–1.11; for PED attendance, 2% per month, IRR 1.02, 95%CI 0.97–1.08).

To better understand this trend, we differentiated between acute and chronic respiratory conditions in hospitalizations, considering primary and secondary diagnoses. As evident in Supplementary Table S11 and Supplementary Figure S5, the overall trend is mainly determined by acute conditions.

#### 3.2.3 Symptoms, signs and ill-defined conditions

For this wide category, consisting in nonspecific abnormal findings and unknown causes of morbidity and mortality, the new data relative to PE (Supplementary Figure S4A; Supplementary Table S10) confirmed the overlapping time series with overall PED attendance (Figure 3B) highlighted in our previous paper (16). Following the important reduction recorded in the two pandemic phases (level change, −81% in SC and −65% in MM), in PE a non-significant decrease compared to PC was observed (level change, IRR 0.77, 95%CI 0.58–1.02), with PED attendance rates reaching pre-pandemic levels only in the first months of 2023 (slope change, 1% per month, IRR 1.01, 95%CI 0.98–1.04).

#### 3.2.4 Injury and poisoning

Rates for this category (Supplementary Figure S4B; Supplementary Table S10), which represents almost one-third of all PED visits, after an initial rapid decline and a subsequent increase in trend during the end of SC and throughout MM, appeared to stabilize in PE, and returned to pre-pandemic levels (level change, IRR 0.89, 95%CI 0.65–1.22). A return to the recurring seasonal trends was also noted with higher rates in spring and summer.

## 4 Discussion

This study aimed to examine the consequences on the utilisation of paediatric health services in Italy after the lifting of COVID-19 pandemic restrictions. To achieve this, we conducted an interrupted time series analysis using data from the health information system on pediatric hospitalisations and PED admissions. Our findings provide valuable insights. First, hospitalisation rates, which had sharply declined during the lockdown and then had undergone a nearly constant slight increase—reaching pre-pandemic levels only after approximately 18 months—rose by more than 20% in the first post-emergency year compared to pre-pandemic levels. In contrast, PED visits, after an initial drastic reduction and a sustained period of reduced utilization throughout the emergency, returned to pre-pandemic rates. These findings suggest that the effects of the pandemic on pediatric emergency care largely dissipated following the end of NPI implementation. Conversely, the increase in hospitalization rates may be at least partially explained by deferred care during the pandemic, particularly for elective and non-urgent procedures (17).

Noteworthy in this regard is the disruption in primary care that occurred during the pandemic, which may have heavily impacted patient health, especially for more vulnerable populations, e. g. more long-term complications for chronic patients owing to missed care, and increases in cancer rates and mortality as a result of missed screenings (18). Other evidence appears to support this hypothesis. A systematic review investigating the impact of COVID-19-related disruptions on clinical outcomes in people with diabetes, including 138 studies that compared prepandemic with pandemic periods (19), found consistent increases in all-cause mortality and diabetes-related admissions to pediatric intensive care units.

To better understand the consequences on health care service utilization in the post-emergency period, we also analyzed trends of individual frequent diagnostic categories, which we had previously identified as exhibiting peculiar patterns (15, 16). Among these, one of the most striking findings concerned hospital admissions for “Mental Disorders,” which warrant extended discussion. Our data revealed that after the emergency, despite a slight decreasing trend, hospitalization rates for this category still averaged more than double the pre-pandemic levels. The steep increases we observed during the pandemic period, and the extremely elevated rates detected one full year after the lifting of NPIs suggest that the pandemic had a severe impact on pediatric mental health, particularly for adolescent girls (12 to 17 years), who were disproportionately affected, exhibiting hospitalization rates in the post-emergency year four times higher than before the outbreak. This finding confirms that female teenagers were the group most severely affected in terms of mental health during the pandemic (20–22), and thus represent the highest-risk group for mental health issues. These gender differences may be explained by several interrelated mechanisms. Psychosocial stressors linked to the pandemic, such as social isolation, disruption of school routines, and increased use of social media, have been associated with heightened risk of anxiety, depression, and eating disorders, conditions more prevalent in females. Biological factors, including hormonal fluctuations during puberty, may further predispose adolescent girls to internalizing disorders. Additionally, girls might be more likely to seek help or to be referred to specialist services, contributing to higher hospitalization rates. These combined factors could underlie the nearly fourfold increase in mental health admissions among adolescent females in the post-emergency period, contrasting with the non-significant change observed in males. Insights from this and future research should inform strategies to prepare in the event of future health crises, particularly targeting individuals at greatest risk (22). These strategies should include innovative models of care that bridge community, acute, and specialist mental health provision (22). Telemedicine offers a promising avenue to expand access to mental health services (23, 24); however, its implementation must ensure equity, accessibility, and quality of care (25, 26). A hybrid approach combining in-person consultations with virtual care could help address the increased demand while maintaining a high standard of care. Furthermore, proactive policies should be developed to identify at-risk populations early and provide timely mental health support to mitigate the long-term effects of the crisis.

Another diagnostic category examined in our previous work was “Respiratory Disorders,” selected due to its high hospitalization frequency and considerable decline during the pandemic. In the post-emergency year, hospitalization rates returned to pre-pandemic levels, and displayed an upward trend. The resurgence of respiratory diseases after the lifting of restrictions suggests that the pandemic has temporarily altered the epidemiology of common pediatric infections, and is likely attributable to the phenomenon of ‘immune debt’. Reduced exposure to common respiratory pathogens during the prolonged implementation of non-pharmaceutical interventions may have led to decreased population immunity, particularly among children. As restrictions were lifted, susceptible cohorts were suddenly exposed to circulating respiratory viruses, leading to a surge in infections. This phenomenon, sometimes called the ‘boomerang effect’ (27) has been documented in other settings (14, 27–29) and aligns with the steady increase in respiratory hospitalizations and PED visits detected in our data. highlight the importance of ongoing surveillance and potential adaptations in vaccination strategies. Anticipating seasonal immunization campaigns or adjusting recommendations based on emerging epidemiological patterns could help mitigate the impact of such rebounds in the future.

Our previous work also investigated emergency care utilization for problems classified as “Symptoms, Signs and Ill-Defined Conditions.” The sharp drop observed during the pandemic in this category, which predominantly includes milder, non-urgent complaints, had led us to speculate that a shift in parental health-seeking behavior may have occurred during the health crisis, leading to improved awareness of the actual need for emergency care, and thus resulting in more appropriate PED utilization (16). However, the return to pre-pandemic rates after NPI lifting seems to suggest that other factors, such as fear of contagion, anti-epidemic policies, and increased telemedicine use, may have been the main drivers of these changes (30). This finding emphasizes the need to strengthen primary pediatric care and community-based health services to optimize emergency department utilization. The implementation of advanced triage strategies, including virtual pediatric consultations before emergency visits, could improve patient flow management and reduce unnecessary hospital burden.

This study has some limitations. Firstly, as pointed out in our previous work, we relied on data that were extracted from hospital databases and were not collected prospectively for this research, or for epidemiologic purposes. However, this should not affect interpretation of findings, since we assume data quality to be similar throughout the entire study period, in all phases under comparison. Secondly, due to strict privacy policies, we were not able to discriminate between new versus recurrent cases. This distinction may be of relevance, especially since evidence points to significant increases in the frequency of hospital readmissions during the pandemic period, particularly for mental disorders (31, 32). Thirdly, due to limitations of the data sources, our analysis could only stratified by sex and age, without collecting detailed demographics or socioeconomic variables. This would be required to obtain a comprehensive assessment of the differential impacts of the pandemic across population subgroups, especially the more vulnerable. Finally, a limitation specifically pertaining to the present study concerns the length of the observation period, which is restricted to a single year following the official end of the COVID-19 emergency. A longer follow-up would have allowed us to assess whether the trends we identified persist, evolve, or diminish over time, providing a more solid and clearer picture of long-term impacts.

## 5 Conclusion

Overall, this study provides valuable insights into the evolving landscape of pediatric healthcare utilization in the aftermath of the COVID-19 pandemic. Notably, our findings show that 1 year after the end of the emergency, hospital admission rates were still significantly above pre-pandemic values, and indicate a striking four-fold increase in mental health-related hospital admissions among adolescent girls compared to before the outbreak. The observed trends underscore the need for proactive policies to prepare for future health crises, with particular attention to vulnerable populations, including children and adolescents. Investing in telemedicine, reinforcing community-based healthcare, and adapting vaccination strategies will be essential steps in building a resilient pediatric healthcare system capable of addressing both the immediate and long-term effects of the pandemic. At the same time, efforts should be made to encourage appropriate timely health-seeking behavior, particularly among vulnerable populations, to prevent more severe consequences in the future. Continued monitoring of these trends will be crucial to inform policy decisions and ensure the delivery of high-quality, equitable pediatric healthcare services in the years to come.

## Data Availability

The data analyzed in this study is subject to the following licenses/restrictions: the raw data supporting the conclusions of this article will be made available by the authors upon a motivated request to the corresponding author. Requests to access these datasets should be directed to Caterina Caminiti, ccaminiti@ao.pr.it.

## Group member of [Emilia-Romagna Paediatric COVID-19 Network]

Francesca Diodati, Denisa Balan: Clinical and Epidemiological Research Unit, University Hospital of Parma, Parma, Italy; Angela Miniaci, Luca Bertelli: Pediatric Clinic, IRCCS Azienda Ospedaliera Universitaria di Bologna, Bologna, Italy; Giovanni Biserni: Pediatric Emergency Unit, IRCCS Azienda Ospedaliera Universitaria di Bologna, Bologna, Italy; Angela Troisi, Alessandra Iacono: Pediatrics and Neonatology Unit, Ravenna Hospital, AUSL Romagna, Ravenna, Italy; Federico Bonvicini, Domenico Bartolomeo, Andrea Trombetta: Paediatrics Unit, Santa Maria Nuova Hospital, AUSL-IRCCS of Reggio Emilia, Reggio Emilia, Italy; Tommaso Zini: Pediatrics Unit, Department of Medical and Surgical Sciences of Mothers, Children and Adults, University of Modena and Reggio Emilia, Modena, Italy; Nicoletta de Paulis: Pediatrics and Neonatology Unit, Guglielmo da Saliceto Hospital, Piacenza, Italy; Cristina Forest: Pediatric Clinic, University of Ferrara, Ferrara, Italy; Battista Guidi: Pediatric Unit, Pavullo Hospital, AUSL Modena, Pavullo, Italy; Francesca Di Florio: Paediatrics Unit, Maggiore Hospital, Bologna, Italy; Francesco Accomando: Pediatric Unit, G.B. Morgagni—L. Pierantoni Hospital, AUSL Romagna, Forlì; Greta Ramundo, Alberto Argentiero: Pediatric Clinic, University Hospital, Department of Medicine and Surgery, University of Parma, Parma, Italy.
