# Supplementary material for: Pediatric healthcare service utilization after the end of COVID-19 state of emergency in Northern Italy: a 6-year quasi-experimental study using interrupted time-series analysis
Source: Front Public Health. 2025 Aug 21;13:1575047. doi: 10.3389/fpubh.2025.1575047 (PMC12408626; doi:10.3389/fpubh.2025.1575047)
Supplement: Supplementary file 1 [file Table_1.docx]

**Supplementary tables**

Supplementary Table S1: Annual standardized hospitalization rates (95%CI) x 100,000 (pop EU 2020), by primary diagnosis and individual phase

Supplementary Table S2: Monthly standardized PED attendance incidence rates (95%CI) x 100,000 (pop EU 2020) by diagnosis and individual phase.

Supplementary Table S3: Monthly hospitalization and PED attendance time trend rates (HRR and IRR, respectively,and corresponding 95%CIs) for each individual phase, obtained with Interrupted time-series analysis.

Supplementary Table S4: Interrupted time series analysis results on hospitalization rates for Mental disorders, by sex.

Supplementary Table S5: Interrupted time series analysis results on PED attendance rates for Mental disorders, by sex.

Supplementary Table S6: Interrupted time series analysis results on hospitalization rates for Mental disorders, by age category.

Supplementary Table S7: Interrupted time series analysis results on PED attendance rates for Mental disorders, by age category.

Supplementary Table S8: Interrupted time series analysis results on PED attendance rates for Mental disorders, in adolescents (12-17y), by sex.

Supplementary Table S9: Interrupted time-series analysis results on hospitalizations and PED attendances rates for Diseases of the Respiratory System.

Supplementary Table S10: Interrupted time-series analysis results on PED attendances rates for Symptoms, Signs and Ill-defined Conditions and for Injury and Poisoning.

Supplementary Table S11: Interrupted time-series analysis results on hospitalizations rates for Acute and Chronic Diseases of the Respiratory System.

| Supplementary Table S1. Annual standardized hospitalization rates (95%CI) x 100,000 (pop EU 2020) and total number, by primary diagnosis and single phase of the study period | | | | | | |
| --- | --- | --- | --- | --- | --- | --- |
|  | **PC**  **(Mar 1, 2017-**  **Feb 28, 2020)** | **SC**  **(Mar 1, 2020-**  **Sep 30, 2020)** | **MM**  **(Oct 1, 2020-**  **Mar 30, 2022)** | **PE**  **(Apr 1, 2022-**  **Mar 30, 2023)** | **Total number**  **(Mar 1, 2017-**  **Mar 30, 2023)** |  |
|  | *(n=54,218)* | *(n=6,737)* | *(n=21,973)* | *(n=21,155)* | *(N=104,083)* |  |
| Infectious and Parasitic Diseases (001-139) | 222 (209-235) | 120 (110-130) | 162 (150-174) | 263 (248-278) | 5,790 (5.6%) |  |
| Neoplasms (140-239) | 63 (56-70) | 62 (55-69) | 81 (73-90) | 149 (138-160) | 2,382 (2.3%) |  |
| Endocrine, Nutritional & Metabolic & Immunity Disorders (240-279) | 99 (89-107) | 68 (61-75) | 98 (90-107) | 154 (143-166) | 3,033 (2.9%) |  |
| Diseases of the Blood and Blood-forming Organs (280-289) | 98 (89-107) | 62 (55-69) | 63 (56-70) | 104 (95-114) | 2,537 (2.4%) |  |
| Mental Disorders (290-319) | 58 (51-65) | 41 (35-46) | 95 (86-103) | 147 (136-157) | 2,427 (2.3%) |  |
| Diseases of the Nervous System and Sense Organs (320-389) | 233 (220-247) | 147 (137-158) | 203 (191-216) | 268 (253-283) | 6,549 (6.3%) |  |
| Diseases of the Circulatory System (390-459) | 55 (48-62) | 33 (28-39) | 52 (46-59) | 80 (72-88) | 1,672 (1.6%) |  |
| Diseases of the Respiratory System (460-519) | 751 (727-776) | 204 (191-217) | 454 (434-473) | 778 (752-804) | 17,931 (17.2%) |  |
| Diseases of the Digestive System (520-579) | 298 (283-313) | 228 (215-242) | 277 (262-291) | 400 (382-418) | 8,981 (8.6%) |  |
| Diseases of the Genitourinary System (580-629) | 193 (181-205) | 136 (126-147) | 159 (147-170) | 260 (246-275) | 5,537 (5.3%) |  |
| Complications of Pregnancy, Childbirth, & Puerperium (630-677) | 9 (6-11) | 5 (3-7) | 6 (4-9) | 15 (11-18) | 262 (0.3%) |  |
| Diseases of the Skin and Subcutaneous Tissue (680-709) | 63 (56-70) | 34 (29-39) | 52 (45-58) | 94 (85-102) | 1,836 (1.8%) |  |
| Dis. of the Musculoskeletal System & Connective Tissue (710-739) | 97 (88-106) | 53 (47-60) | 71 (64-79) | 107 (98-116) | 2,650 (2.6%) |  |
| Congenital Anomalies (740-759) | 201 (188-214) | 157 (146-168) | 172 (160-184) | 316 (299-332) | 5,885 (5.7%) |  |
| Certain Conditions originating in the Perinatal Period (760-779) | 482 (463-501) | 470 (451-490) | 451.3 (432-471) | 477 (457-497) | 12,647 (12.2%) |  |
| Symptoms, Signs and Ill-defined Conditions (780-799) | 311 (295-326) | 195 (182-208) | 257.0 (242-272) | 373 (356-391) | 8,517 (8.2%) |  |
| Injury and Poisoning (800-999) | 330 (314-346) | 299 (284-314) | 291 (276-306) | 353 (337-370) | 9,561 (9.2%) |  |
| Abbreviations: PC= Pre-COVID19 phase; SC= School closure phase; MM=Mitigation Measures phase; PE=Post-emergency phase; 95%CI: 95% confidence interval | | | | | | |

| Supplementary Table S2. Monthly standardized PED attendance incidence rates (95%CI) x 100,000 (pop EU 2020) and total number, by type of diagnosis and single phase of the study period | | | | | |
| --- | --- | --- | --- | --- | --- |
|  | **PC**  **(Mar 1, 2017-**  **Feb 28, 2020)** | **SC**  **(Mar 1, 2020-**  **Sep 30, 2020)** | **MM**  **(Oct 1, 2020-**  **Mar 30, 2022)** | **PE**  **(Apr 1, 2022-**  **Mar 30, 2023)** | **Total number**  **(Mar 1, 2017-**  **Mar 30, 2023)** |
|  | *(n=506,496)* | *(n=41,714)* | *(n=153,425)* | *(n=157,127)* | *(N=858,762)* |
| Infectious and Parasitic Diseases (001-139) | 161 (150-173) | 39 (34-45) | 72 (64-80) | 158 (146-170) | 43,643 (5.1%) |
| Neoplasms (140-239) | 1 (0-2) | 1 (0-1) | 0 (0-1) | 1 (0-2) | 483 (0.1%) |
| Endocrine, Nutritional & Metabolic & Immunity Disorders (240-279) | 8 (6-11) | 4 (2-6) | 6 (4-9) | 9 (7-12) | 2,532 (0.3%) |
| Diseases of the Blood and Blood-forming Organs (280-289) | 6 (4-8) | 4 (2-5) | 5 (3-7) | 7 (5-10) | 2,062 (0.3%) |
| Mental Disorders (290-319) | 22 (18-26) | 16 (12-19) | 23 (18-27) | 24 (20-29) | 8,003 (1.0%) |
| Diseases of the Nervous System and Sense Organs (320-389) | 184 (172-197) | 64 (57-71) | 94 (86-103) | 193 (180-205) | 53,135 (6.2%) |
| Diseases of the Circulatory System (390-459) | 9 (7-12) | 6 (3-8) | 6 (4-9) | 7 (5-9) | 2,740 (0.3%) |
| Diseases of the Respiratory System (460-519) | 441 (422-460) | 92 (83-100) | 234 (220-248) | 506 (485-527) | 124,239 (14.5%) |
| Diseases of the Digestive System (520-579) | 114 (104-123) | 47 (41-54) | 67 (60-74) | 99 (90-108) | 32,646 (3.8%) |
| Diseases of the Genitourinary System (580-629) | 49 (42-55) | 30 (25-35) | 41 (36-47) | 56 (49-63) | 16,129 (1.9%) |
| Complications of Pregnancy, Childbirth, & Puerperium (630-677) | 1 (0-3) | 1 (0-2) | 2 (1-3) | 3 (1-5) | 662 (0.1%) |
| Diseases of the Skin and Subcutaneous Tissue (680-709) | 89 (81-98) | 35 (29-40) | 43 (37-49) | 86 (78-95) | 25,233 (2.9%) |
| Dis. of the Musculoskeletal System & Connective Tissue (710-739) | 63 (56-70) | 30 (25-35) | 43 (37-49) | 58 (51-65) | 19,434 (2.3%) |
| Congenital Anomalies (740-759) | 8 (5-10) | 3 (2-5) | 3 (1-5) | 5 (3-7) | 1,832 (0.2%) |
| Certain Conditions originating in the Perinatal Period (760-779) | 4 (2-6) | 2 (1-3) | 3 (1-5) | 4 (2-5) | 1,175 (0.1%) |
| Symptoms, Signs and Ill-defined Conditions (780-799) | 810 (785-835) | 307 (292-323) | 499 (479-520) | 761 (736-786) | 234,540 (27.3%) |
| Injury and Poisoning (800-999) | 829 (804-854) | 504 (484-524) | 579 (558-600) | 722 (698-747) | 256,370 (29.9%) |
| Abbreviations: PED= pediatric emergency department; PC= Pre-COVID19 phase; SC= School closure phase; MM=Mitigation Measures phase; PE=Post-emergency phase; 95%CI: 95% confidence interval | | | | | |

| Supplementary Table S3: Monthly hospitalization and PED attendance time trend rates (HRR and IRR, respectively, and corresponding 95%CIs) for each individual phase, obtained with Interrupted time-series analysis. | | | | |
| --- | --- | --- | --- | --- |
|  | **PC**  **(Mar 1, 2017-**  **Feb 28, 2020)** | **SC**  **(Mar 1, 2020-**  **Sep 30, 2020)** | **MM**  **(Oct 1, 2020-**  **Mar 30, 2022)** | **PE**  **(Apr 1, 2022-**  **Mar 30, 2023)** |
| *Any disease:* |  |  |  |  |
| Hospitalization | 1.00 (0.99-1.01) | 1.09 (1.04-1.13)*** | 1.03 (1.01-1.04)*** | 1.01 (1.00-1.02)* |
| PED attendance | 1.00 (0.99-1.01) | 1.20 (1.10-1.31)*** | 1.05 (1.03-1.07)*** | 1.01 (0.99-1.03) |
|  |  |  |  |  |
| *Mental Disorders* |  |  |  |  |
| Hospitalization | 1.00 (0.99-1.01) | 1.09 (1.02-1.17)* | 1.03 (1.00-1.07)* | 0.97 (0.95-0.99)** |
| PED attendance | 1.01 (1.00-1.01)** | 1.21 (1.08-1.35)*** | 1.03 (1.01-1.05)** | 0.99 (0.96-1.02) |
|  |  |  |  |  |
| *Diseases of the Respiratory System* |  |  |  |  |
| Hospitalization | 1.01 (0.99-1.02) | 1.05 (0.87-1.27) | 1.07 (1.02-1.12)** | 1.12 (1.05-1.19)*** |
| PED attendance | 1.01 (0.99-1.02) | 1.25 (0.97-1.60) | 1.10 (1.05-1.16)*** | 1.08 (1.01-1.14)* |
|  |  |  |  |  |
| *Symptoms, Signs and Ill-defined Conditions* |  |  |  |  |
| PED attendance | 1.00 (0.99-1.01) | 1.14 (1.08-1.19)*** | 1.05 (1.03-1.07)*** | 1.02 (0.99-1.06) |
|  |  |  |  |  |
| *Injury and Poisoning* |  |  |  |  |
| PED attendance | 1.00 (0.99-1.01) | 1.23 (1.08-1.39)*** | 1.02 (0.99-1.05) | 0.96 (0.93-0.99)** |
|  |  |  |  |  |
| Abbreviations: PC= Pre-COVID19 phase; SC= School closure phase; MM=Mitigation Measures phase; PE=Post-emergency phase; 95%CI: 95% confidence interval; HRR= Hospitalization Rate Ratio; IRR= Incidence Rate Ratio. Notes: *p<0.05, **p<0.01, ***p<0.001. | | | | |

| Supplementary Table S4: Interrupted time-series analysis results on hospitalization rates for Mental disorders, by sex. | | | | | | |
| --- | --- | --- | --- | --- | --- | --- |
|  | **Males** | | | **Females** | | |
|  | ***HRR*** | ***95%CI*** | ***p-Value*** | ***HRR*** | ***95%CI*** | ***p-Value*** |
| *Level change^a^* |  |  |  |  |  |  |
| SC vs. PC | 0.53 | 0.26-1.08 | 0.082 | 0.46 | 0.21-0.97 | 0.042 |
| MM vs. PC | 0.75 | 0.48-1.17 | 0.206 | 1.49 | 1.01-2.19 | 0.042 |
| PE vs. PC | 2.44 | 1.34-4.44 | 0.004 | 2.72 | 1.53-4.84 | 0.001 |
|  |  |  |  |  |  |  |
| *Slope change^b^* |  |  |  |  |  |  |
| SC vs. PC | 1.12 | 0.97-1.29 | 0.139 | 1.14 | 0.98-1.33 | 0.085 |
| MM vs. PC | 1.04 | 1.00-1.07 | 0.026 | 1.03 | 1.01-1.06 | 0.011 |
| PE vs. PC | 0.95 | 0.91-1.00 | 0.030 | 0.98 | 0.94-1.03 | 0.420 |
|  |  |  |  |  |  |  |
| *Time trend^c^* | 1.00 | 0.99-1.02 | 0.409 | 1.00 | 0.99-1.01 | 0.738 |
|  |  |  |  |  |  |  |
| *Season* |  |  |  |  |  |  |
| Summer | 1.00 |  |  | 1.00 |  |  |
| Winter | 1.24 | 0.98-1.57 | 0.068 | 1.08 | 0.88-1.34 | 0.447 |
| Spring | 1.18 | 0.95-1.48 | 0.143 | 1.20 | 0.98-1.47 | 0.073 |
| Autumn | 1.18 | 0.94-1.48 | 0.153 | 1.08 | 0.88-1.32 | 0.458 |
|  |  |  |  |  |  |  |
| ^a^ Level change refers to an abrupt level change of the Incidence rate between the periods; ^b^ Slope change refers to slope change of the incidence rate over time between the periods. ^c^ Time trend refers to the change of Incidence rate associated with a time unit increase. Abbreviations: HRR= Hospitalization Rate Ratio; PC= Pre-COVID19 phase; SC= School closure phase; MM=Mitigation Measures phase; PE=Post Emergency phase; 95%CI: 95% confidence interval. Pseudo R2=0.29 for Males model, 0.39 for Females model. | | | | | | |

| Supplementary Table S5: Interrupted time-series analysis results on PED attendance rates for Mental disorders, by sex. | | | | | | |
| --- | --- | --- | --- | --- | --- | --- |
|  | **Males** | | | **Females** | | |
|  | ***IRR*** | ***95%CI*** | ***p-Value*** | ***IRR*** | ***95%CI*** | ***p-Value*** |
| *Level change^a^* |  |  |  |  |  |  |
| SC vs. PC | 0.31 | 0.17-0.54 | <0.001 | 0.24 | 0.15-0.40 | <0.001 |
| MM vs. PC | 0.73 | 0.54-0.99 | 0.045 | 0.66 | 0.52-0.86 | 0.002 |
| PE vs. PC | 0.65 | 0.41-1.02 | 0.060 | 0.92 | 0.64-1.32 | 0.657 |
|  |  |  |  |  |  |  |
| *Slope change^b^* |  |  |  |  |  |  |
| SC vs. PC | 1.15 | 1.03-1.29 | 0.011 | 1.23 | 1.12-1.35 | <0.001 |
| MM vs. PC | 1.00 | 0.98-1.03 | 0.916 | 1.03 | 1.01-1.05 | <0.001 |
| PE vs. PC | 1.01 | 0.97-1.05 | 0.750 | 0.99 | 0.96-1.02 | 0.571 |
|  |  |  |  |  |  |  |
| *Time trend^c^* | 1.01 | 1.00-1.02 | 0.015 | 1.01 | 1.00-1.01 | 0.037 |
|  |  |  |  |  |  |  |
| *Season* |  |  |  |  |  |  |
| Summer | 1.00 |  |  | 1.00 |  |  |
| Winter | 0.83 | 0.70-0.97 | 0.022 | 0.99 | 0.87-1.13 | 0.900 |
| Spring | 0.93 | 0.79-1.09 | 0.367 | 1.09 | 0.96-1.25 | 0.186 |
| Autumn | 0.79 | 0.67-0.93 | 0.005 | 0.92 | 0.81-1.05 | 0.225 |
|  |  |  |  |  |  |  |
| ^a^ Level change refers to an abrupt level change of the Incidence rate between the periods; ^b^ Slope change refers to slope change of the incidence rate over time between the periods. ^c^ Time trend refers to the change of Incidence rate associated with a time unit increase. Abbreviations: Abbreviations: PED= Pediatric Emergency Department; IRR= Incidence Rate Ratio; PC= Pre-COVID19 phase; SC= School closure phase; MM=Mitigation Measures phase; PE=Post Emergency phase; IRR= Incidence rate ratio; 95%CI: 95% confidence interval. Pseudo R2=0.13 for Males model, 0.24 for Females model. | | | | | | |

| Supplementary Table S6: Interrupted time-series analysis results on hospitalization rates for Mental disorders, by age category. | | | | | | | | | |
| --- | --- | --- | --- | --- | --- | --- | --- | --- | --- |
|  | **0-5y** | | | **6-11y** | | | **12-17y** | | |
|  | ***HRR*** | ***95%CI*** | ***p-Value*** | ***HRR*** | ***95%CI*** | ***p-Value*** | ***HRR*** | ***95%CI*** | ***p-Value*** |
| *Level change^a^* |  |  |  |  |  |  |  |  |  |
| SC vs. PC | 0.49 | 0.22-1.11 | 0.087 | 0.65 | 0.29-1.49 | 0.312 | 0.44 | 0.22-0.87 | 0.019 |
| MM vs. PC | 0.65 | 0.39-1.09 | 0.101 | 0.92 | 0.55-1.53 | 0.747 | 1.45 | 1.02-2.05 | 0.038 |
| PE vs. PC | 2.14 | 1.11-4.12 | 0.024 | 2.95 | 1.47-5.92 | 0.002 | 2.69 | 1.59-4.57 | <0.001 |
|  |  |  |  |  |  |  |  |  |  |
| *Slope change^b^* |  |  |  |  |  |  |  |  |  |
| SC vs. PC | 1.13 | 0.95-1.34 | 0.167 | 1.04 | 0.87-1.25 | 0.653 | 1.16 | 1.02-1.33 | 0.030 |
| MM vs. PC | 1.05 | 1.01-1.09 | 0.007 | 1.03 | 0.99-1.06 | 0.152 | 1.03 | 1.01-1.05 | 0.013 |
| PE vs. PC | 0.99 | 0.95-1.05 | 0.818 | 0.93 | 0.88-0.98 | 0.011 | 0.97 | 0.93-1.01 | 0.095 |
|  |  |  |  |  |  |  |  |  |  |
| *Time trend^c^* | 1.00 | 0.99-1.01 | 0.766 | 1.00 | 0.99-1.01 | 0.817 | 1.00 | 0.99-1.01 | 0.972 |
|  |  |  |  |  |  |  |  |  |  |
| *Season* |  |  |  |  |  |  |  |  |  |
| Summer | 1.00 |  |  | 1.00 |  |  | 1.00 |  |  |
| Winter | 1.35 | 1.04-1.77 | 0.026 | 1.29 | 0.99-1.69 | 0.061 | 1.00 | 0.85-1.25 | 0.756 |
| Spring | 1.47 | 1.15-1.89 | 0.003 | 1.23 | 0.95-1.60 | 0.119 | 1.08 | 0.90-1.30 | 0.399 |
| Autumn | 1.20 | 0.92-1.55 | 0.173 | 1.10 | 0.84-1.43 | 0.509 | 1.10 | 0.91-1.31 | 0.322 |
|  |  |  |  |  |  |  |  |  |  |
| ^a^ Level change refers to an abrupt level change of the Incidence rate between the periods; ^b^ Slope change refers to slope change of the incidence rate over time between the periods. ^c^ Time trend refers to the change of Incidence rate associated with a time unit increase. Abbreviations: HRR=Hospitalization Rate Ratio; PC= Pre-COVID19 phase; SC= School closure phase; MM=Mitigation Measures phase; PE=Post Emergency phase; 95%CI: 95% confidence interval. Pseudo R2=0.23 for 0-5y model, 0.16 for 6-11 model, 0.43 for 12-17y model. | | | | | | | | | |

| Supplementary Table S7: Interrupted time-series analysis results on PED attendance rates for Mental disorders, by age category. | | | | | | | | | |
| --- | --- | --- | --- | --- | --- | --- | --- | --- | --- |
|  | **0-5y** | | | **6-11y** | | | **12-17y** | | |
|  | ***IRR*** | ***95%CI*** | ***p-Value*** | ***IRR*** | ***95%CI*** | ***p-Value*** | ***IRR*** | ***95%CI*** | ***p-Value*** |
| *Level change^a^* |  |  |  |  |  |  |  |  |  |
| SC vs. PC | 0.33 | 0.15-0.74 | 0.007 | 0.33 | 0.14-0.73 | 0.007 | 0.25 | 0.15-0.41 | <0.001 |
| MM vs. PC | 0.57 | 0.36-0.90 | 0.015 | 0.87 | 0.57-1.34 | 0.525 | 0.68 | 0.52-0.88 | 0.003 |
| PE vs. PC | 0.70 | 0.37-1.31 | 0.263 | 0.91 | 0.49-1.68 | 0.761 | 0.79 | 0.54-1.15 | 0.223 |
|  |  |  |  |  |  |  |  |  |  |
| *Slope change^b^* |  |  |  |  |  |  |  |  |  |
| SC vs. PC | 1.10 | 0.94-1.30 | 0.230 | 1.12 | 0.95-1.32 | 0.196 | 1.22 | 1.11-1.35 | <0.001 |
| MM vs. PC | 1.01 | 0.98-1.05 | 0.564 | 1.00 | 0.97-1.03 | 0.959 | 1.03 | 1.01-1.05 | 0.009 |
| PE vs. PC | 0.99 | 0.93-1.04 | 0.614 | 1.01 | 0.96-1.06 | 0.751 | 1.00 | 0.96-1.03 | 0.852 |
|  |  |  |  |  |  |  |  |  |  |
| *Time trend^c^* | 1.01 | 1.00-1.02 | 0.022 | 1.01 | 0.99-1.02 | 0.271 | 1.01 | 1.00-1.01 | 0.071 |
|  |  |  |  |  |  |  |  |  |  |
| *Season* |  |  |  |  |  |  |  |  |  |
| Summer | 1.00 |  |  | 1.00 |  |  | 1.00 |  |  |
| Winter | 0.95 | 0.74-1.20 | 0.645 | 1.04 | 0.83-1.32 | 0.720 | 0.88 | 0.77-1.01 | 0.075 |
| Spring | 1.13 | 0.90-1.42 | 0.285 | 1.21 | 0.97-1.51 | 0.091 | 0.96 | 0.84-1.10 | 0.569 |
| Autumn | 0.91 | 0.72-1.15 | 0.439 | 0.84 | 0.66-1.06 | 0.146 | 0.87 | 0.76-0.99 | 0.036 |
|  |  |  |  |  |  |  |  |  |  |
| ^a^ Level change refers to an abrupt level change of the Incidence rate between the periods; ^b^ Slope change refers to slope change of the incidence rate over time between the periods. ^c^ Time trend refers to the change of Incidence rate associated with a time unit increase. Abbreviations: PED= Pediatric Emergency Department; IRR= Incidence Rate Ratio; PC= Pre-COVID19 phase; SC= School closure phase; MM=Mitigation Measures phase; PE=Post Emergency phase; IRR= Incidence rate ratio; 95%CI: 95% confidence interval. Pseudo R2=0.07 for 0-5y model, 0.10 for 6-11 model, 0.24 for 12-17y model. | | | | | | | | | |

| Supplementary Table S8: Interrupted time-series analysis results on PED attendance rates for Mental disorders, in adolescents (12-17y), by sex. | | | | | | |
| --- | --- | --- | --- | --- | --- | --- |
|  | **Males** | | | **Females** | | |
|  | ***IRR*** | ***95%CI*** | ***p-Value*** | ***IRR*** | ***95%CI*** | ***p-Value*** |
| *Level change^a^* |  |  |  |  |  |  |
| SC vs. PC | 0.26 | 0.13-0.52 | <0.001 | 0.25 | 0.14-0.43 | <0.001 |
| MM vs. PC | 0.70 | 0.48-1.01 | 0.059 | 0.67 | 0.51-0.89 | 0.006 |
| PE vs. PC | 0.61 | 0.35-1.06 | 0.077 | 0.92 | 0.61-1.37 | 0.674 |
|  |  |  |  |  |  |  |
| *Slope change^b^* |  |  |  |  |  |  |
| SC vs. PC | 1.21 | 1.06-1.38 | 0.004 | 1.23 | 1.11-1.36 | <0.001 |
| MM vs. PC | 1.01 | 0.98-1.03 | 0.734 | 1.04 | 1.02-1.06 | <0.001 |
| PE vs. PC | 1.01 | 0.96-1.07 | 0.643 | 0.99 | 0.95-1.03 | 0.548 |
|  |  |  |  |  |  |  |
| *Time trend^c^* | 1.01 | 0.99-1.02 | 0.096 | 1.01 | 0.99-1.01 | 0.176 |
|  |  |  |  |  |  |  |
| *Season* |  |  |  |  |  |  |
| Summer | 1.00 |  |  | 1.00 |  |  |
| Winter | 0.77 | 0.63-0.93 | 0.008 | 0.96 | 0.83-1.11 | 0.578 |
| Spring | 0.85 | 0.70-1.03 | 0.095 | 1.04 | 0.90-1.20 | 0.618 |
| Autumn | 0.79 | 0.65-0.96 | 0.015 | 0.92 | 0.79-1.06 | 0.234 |
|  |  |  |  |  |  |  |
| ^a^ Level change refers to an abrupt level change of the Incidence rate between the periods; ^b^ Slope change refers to slope change of the incidence rate over time between the periods. ^c^ Time trend refers to the change of Incidence rate associated with a time unit increase. Abbreviations: PED= Pediatric Emergency Department; IRR= Incidence Rate Ratio; PC= Pre-COVID19 phase; SC= School closure phase; MM=Mitigation Measures phase; PE=Post Emergency phase; 95%CI: 95% confidence interval. Pseudo R2=0.13 for Males model, 0.22 for Females model. | | | | | | |

| Supplementary Table S9: Interrupted time-series analysis results on hospitalizations and PED attendances rates for Diseases of the Respiratory System. | | | |
| --- | --- | --- | --- |
| **Variable** | **HRR** | **95%CI** | **p-Value** |
|  |  |  |  |
| *Hospitalizations:* |  |  |  |
|  |  |  |  |
| *Level change^a^* |  |  |  |
| SC vs. PC | 0.19 | 0.08-0.47 | <0.001 |
| MM vs. PC | 0.28 | 0.18-0.48 | <0.001 |
| PE vs. PC | 0.63 | 0.35-1.14 | 0.129 |
|  |  |  |  |
| *Slope change^b^* |  |  |  |
| SC vs. PC | 1.17 | 0.97-1.42 | 0.093 |
| MM vs. PC | 1.06 | 1.03-1.09 | <0.001 |
| PE vs. PC | 1.05 | 1.00-1.11 | 0.060 |
|  |  |  |  |
| *Time trend^c^* | 1.00 | 0.99-1.01 | 0.634 |
|  |  |  |  |
| *Season* |  |  |  |
| Summer | 1.00 |  |  |
| Winter | 2.09 | 1.66-2.64 | <0.001 |
| Spring | 1.40 | 1.09-1.79 | 0.008 |
| Autumn | 2.11 | 1.68-2.65 | <0.001 |
|  |  |  |  |
|  |  |  |  |
| *PED attendances:* | **IRR** | **95%CI** | **p-Value** |
|  |  |  |  |
| *Level change^a^* |  |  |  |
| SC vs. PC | 0.07 | 0.02-0.22 | <0.001 |
| MM vs. PC | 0.18 | 0.11-0.29 | <0.001 |
| PE vs. PC | 0.87 | 0.50-1.50 | 0.615 |
|  |  |  |  |
| *Slope change^b^* |  |  |  |
| SC vs. PC | 1.36 | 1.08-1.72 | 0.009 |
| MM vs. PC | 1.09 | 1.05-1.13 | <0.001 |
| PE vs. PC | 1.02 | 0.97-1.08 | 0.353 |
|  |  |  |  |
| *Time trend^c^* | 1.00 | 0.99-1.01 | 0.723 |
|  |  |  |  |
| *Season* |  |  |  |
| Summer | 1.00 |  |  |
| Winter | 1.81 | 1.44-2.27 | <0.001 |
| Spring | 1.36 | 1.07-1.72 | 0.011 |
| Autumn | 2.03 | 1.64-2.52 | <0.001 |
|  |  |  |  |
| ^a^ Level change refers to an abrupt level change of the Incidence rate between the phases; ^b^ Slope change refers to slope change of the incidence rate over time between the phases; ^c^ Time trend refers to the change of Incidence rate associated with a time unit increase. PED=Pediatric Emergency Department; HRR=Hospitalization Rate Ratio; IRR=incidence rate ratio; PC=pre-COVID19 phase; PC=pre-COVID19 phase; SC=School closure phase; MM=Mitigation measures phase; PE=Post-emergency phase; 95%CI: 95% confidence interval. Pseudo R2 for hospitalization rate model=0.69, for PED attendance rate model=0.78. | | | |

| Supplementary Table S10: Interrupted time-series analysis results on PED attendances rates for Symptoms, Signs and Ill-defined Conditions and for Injury and Poisoning. | | | |
| --- | --- | --- | --- |
| **Variable** | **IRR** | **95%CI** | **p-Value** |
|  |  |  |  |
| *Symptoms, Signs and Ill-defined Conditions* | |  |  |
|  |  |  |  |
| *Level change^a^* |  |  |  |
| SC vs. PC | 0.19 | 0.12-0.29 | <0.001 |
| MM vs. PC | 0.35 | 0.28-0.44 | <0.001 |
| PE vs. PC | 0.77 | 0.58-1.02 | 0.071 |
|  |  |  |  |
| *Slope change^b^* |  |  |  |
| SC vs. PC | 1.18 | 1.08-1.29 | <0.001 |
| MM vs. PC | 1.04 | 1.03-1.06 | <0.001 |
| PE vs. PC | 1.01 | 0.98-1.04 | 0.520 |
|  |  |  |  |
| *Time trend^c^* | 1.00 | 0.99-1.01 | 0.281 |
|  |  |  |  |
| *Season* |  |  |  |
| Summer | 1.00 |  |  |
| Winter | 1.25 | 1.11-1.39 | <0.001 |
| Spring | 1.19 | 1.07-1.33 | 0.002 |
| Autumn | 1.27 | 1.14-1.42 | <0.001 |
|  |  |  |  |
|  |  |  |  |
| *Injury and Poisoning* |  |  |  |
|  |  |  |  |
| *Level change^a^* |  |  |  |
| SC vs. PC | 0.24 | 0.15-0.36 | <0.001 |
| MM vs. PC | 0.59 | 0.46-0.76 | <0.001 |
| PE vs. PC | 0.89 | 0.65-1.22 | 0.484 |
|  |  |  |  |
| *Slope change^b^* |  |  |  |
| SC vs. PC | 1.22 | 1.12-1.33 | <0.001 |
| MM vs. PC | 1.03 | 1.01-1.05 | 0.002 |
| PE vs. PC | 1.01 | 0.98-1.04 | 0.469 |
|  |  |  |  |
| *Time trend^c^* | 1.00 | 0.99-1.01 | 0.482 |
|  |  |  |  |
| *Season* |  |  |  |
| Summer | 1.00 |  |  |
| Winter | 0.72 | 0.64-0.81 | <0.001 |
| Spring | 1.10 | 0.99-1.22 | 0.084 |
| Autumn | 0.75 | 0.67-0.85 | <0.001 |
|  |  |  |  |
| ^a^ Level change refers to an abrupt level change of the Incidence rate between the phases; ^b^ Slope change refers to slope change of the incidence rate over time between the phases; ^c^ Time trend refers to the change of Incidence rate associated with a time unit increase. PED=Pediatric Emergency Department; IRR=incidence rate ratio; PC=pre-COVID19 phase; PC=pre-COVID19 phase; SC=School closure phase; MM=Mitigation measures phase; PE=Post-emergency phase; 95%CI: 95% confidence interval. Pseudo R2 for Symptoms, Signs and Ill-defined Conditions model=0.81, for Injury and Poisoning model=0.74. | | | |

| Supplementary Table S11: Interrupted time-series analysis results on hospitalizations rates for Acute and Chronic Diseases of the Respiratory System. | | | |
| --- | --- | --- | --- |
| **Variable** | **IRR** | **95%CI** | **p-Value** |
|  |  |  |  |
| *Acute* | |  |  |
|  |  |  |  |
| *Level change^a^* |  |  |  |
| SC vs. PC | 0.24 | 0.06-1.02 | 0.053 |
| MM vs. PC | 0.18 | 0.08-0.42 | <0.001 |
| PE vs. PC | 0.57 | 0.20-1.63 | 0.296 |
|  |  |  |  |
| *Slope change^b^* |  |  |  |
| SC vs. PC | 1.12 | 0.79-1.56 | 0.530 |
| MM vs. PC | 1.09 | 1.03-1.15 | 0.004 |
| PE vs. PC | 1.07 | 0.98-1.18 | 0.130 |
|  |  |  |  |
| *Time trend^c^* | 1.00 | 0.99-1.02 | 0.775 |
|  |  |  |  |
| *Season* |  |  |  |
| Summer | 1.00 |  |  |
| Winter | 3.39 | 2.18-5.27 | <0.001 |
| Spring | 1.51 | 0.93-2.45 | 0.100 |
| Autumn | 3.28 | 2.13-5.04 | <0.001 |
|  |  |  |  |
|  |  |  |  |
| *Chronic* |  |  |  |
|  |  |  |  |
| *Level change^a^* |  |  |  |
| SC vs. PC | 0.16 | 0.05-0.55 | 0.004 |
| MM vs. PC | 0.42 | 0.24-0.74 | 0.003 |
| PE vs. PC | 1.78 | 0.86-3.69 | 0.122 |
|  |  |  |  |
| *Slope change^b^* |  |  |  |
| SC vs. PC | 1.30 | 1.03-1.64 | 0.026 |
| MM vs. PC | 1.07 | 1.03-1.11 | 0.001 |
| PE vs. PC | 0.96 | 0.90-1.03 | 0.225 |
|  |  |  |  |
| *Time trend^c^* | 1.00 | 0.99-1.01 | 0.560 |
|  |  |  |  |
| *Season* |  |  |  |
| Summer | 1.00 |  |  |
| Winter | 1.54 | 1.16-2.05 | 0.000 |
| Spring | 1.07 | 0.79-1.44 | 0.663 |
| Autumn | 1.52 | 1.15-2.01 | 0.003 |
|  |  |  |  |
| ^a^ Level change refers to an abrupt level change of the Incidence rate between the phases; ^b^ Slope change refers to slope change of the incidence rate over time between the phases; ^c^ Time trend refers to the change of Incidence rate associated with a time unit increase. IRR=incidence rate ratio; PC=pre-COVID19 phase; PC=pre-COVID19 phase; SC=School closure phase; MM=Mitigation measures phase; PE=Post-emergency phase; 95%CI: 95% confidence interval. Pseudo R2 for Acute Respiratory Diseases=0.62, for Chronic Respiratory Diseases=0.23. Note: Acute cases refer to encounters without any chronic respiratory disease diagnosis (“pure” acute). Chronic cases include any encounter with chronic respiratory disease codes listed as either primary or secondary diagnoses. | | | |
