# Supplementary material for: Pediatric healthcare service utilization after the end of COVID-19 state of emergency in Northern Italy: a 6-year quasi-experimental study using interrupted time-series analysis
Source: Front Public Health. 2025 Aug 21;13:1575047. doi: 10.3389/fpubh.2025.1575047 (PMC12408626; doi:10.3389/fpubh.2025.1575047)
Supplement: Supplementary file 3 [file Presentation_2.pptx]

## Slide 1
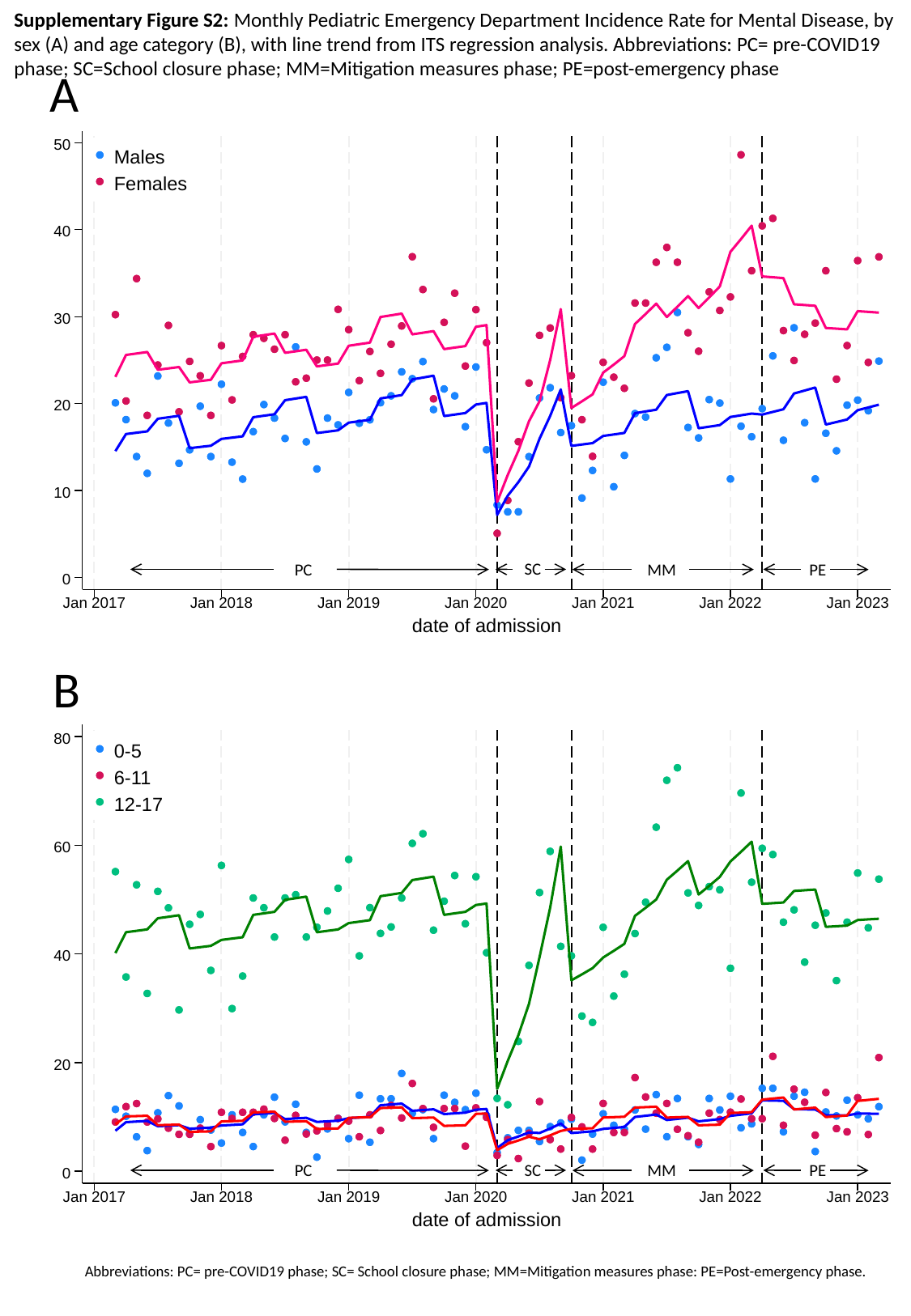

Supplementary Figure S2: Monthly Pediatric Emergency Department Incidence Rate for Mental Disease, by sex (A) and age category (B), with line trend from ITS regression analysis. Abbreviations: PC= pre-COVID19 phase; SC=School closure phase; MM=Mitigation measures phase; PE=post-emergency phase
A
SC
MM
PC
PE
B
SC
MM
PC
PE
Abbreviations: PC= pre-COVID19 phase; SC= School closure phase; MM=Mitigation measures phase: PE=Post-emergency phase.
