# Supplementary material for: Pediatric healthcare service utilization after the end of COVID-19 state of emergency in Northern Italy: a 6-year quasi-experimental study using interrupted time-series analysis
Source: Front Public Health. 2025 Aug 21;13:1575047. doi: 10.3389/fpubh.2025.1575047 (PMC12408626; doi:10.3389/fpubh.2025.1575047)
Supplement: Supplementary file 4 [file Presentation_3.pptx]

## Slide 1
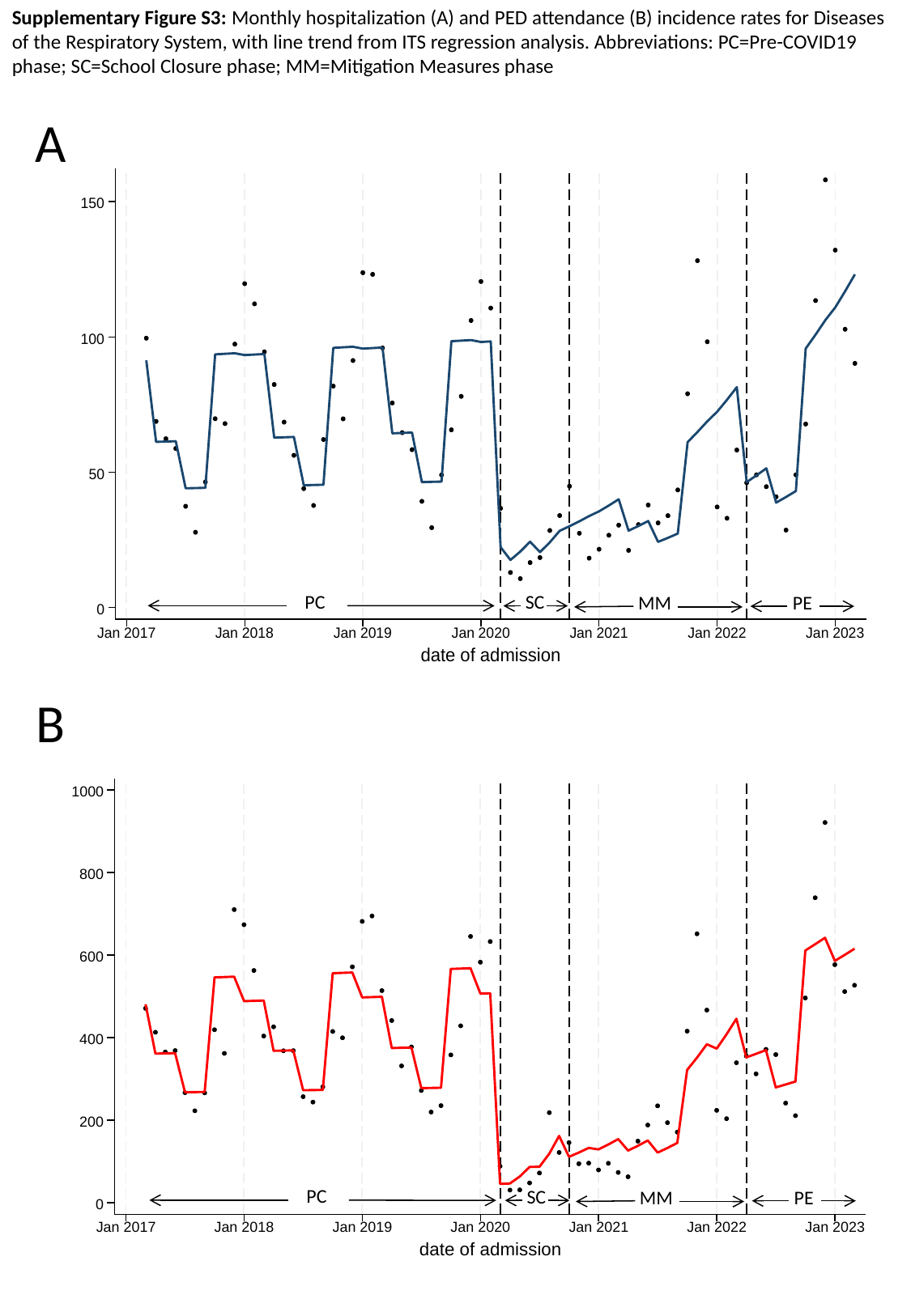

Supplementary Figure S3: Monthly hospitalization (A) and PED attendance (B) incidence rates for Diseases of the Respiratory System, with line trend from ITS regression analysis. Abbreviations: PC=Pre-COVID19 phase; SC=School Closure phase; MM=Mitigation Measures phase
A
PC
SC
MM
PE
B
PC
SC
MM
PE
