# Supplementary material for: Pediatric healthcare service utilization after the end of COVID-19 state of emergency in Northern Italy: a 6-year quasi-experimental study using interrupted time-series analysis
Source: Front Public Health. 2025 Aug 21;13:1575047. doi: 10.3389/fpubh.2025.1575047 (PMC12408626; doi:10.3389/fpubh.2025.1575047)
Supplement: Supplementary file 5 [file Presentation_4.pptx]

## Slide 1
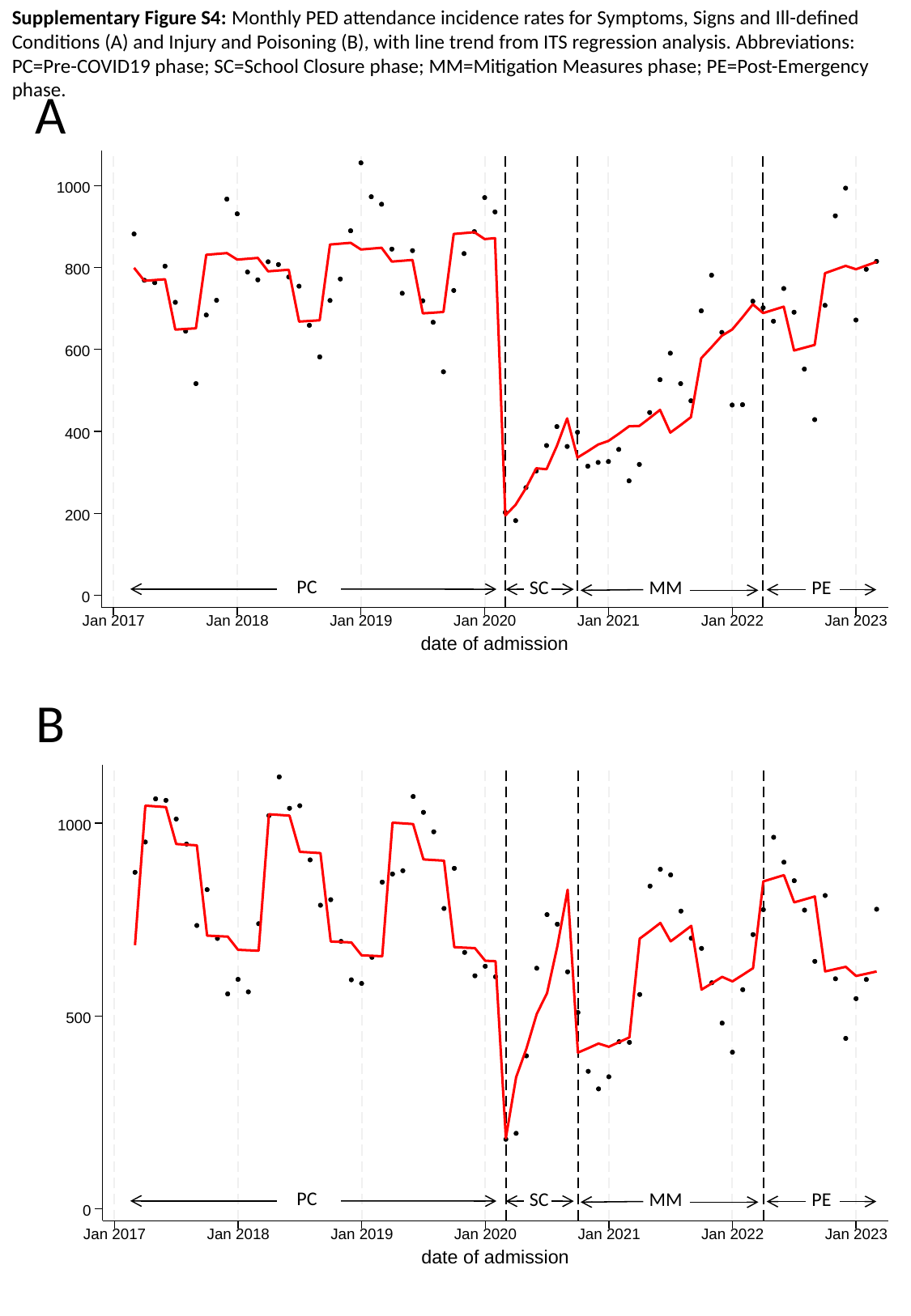

Supplementary Figure S4: Monthly PED attendance incidence rates for Symptoms, Signs and Ill-defined Conditions (A) and Injury and Poisoning (B), with line trend from ITS regression analysis. Abbreviations: PC=Pre-COVID19 phase; SC=School Closure phase; MM=Mitigation Measures phase; PE=Post-Emergency phase.
A
PC
SC
MM
PE
B
PC
SC
MM
PE
